# Measurement of the jet mass in highly boosted $${\mathrm{t}}\overline{\mathrm{t}}$$ events from pp collisions at $$\sqrt{s}=8$$$$\,\text {TeV}$$

**DOI:** 10.1140/epjc/s10052-017-5030-3

**Published:** 2017-07-14

**Authors:** A. M. Sirunyan, A. Tumasyan, W. Adam, E. Asilar, T. Bergauer, J. Brandstetter, E. Brondolin, M. Dragicevic, J. Erö, M. Flechl, M. Friedl, R. Frühwirth, V. M. Ghete, C. Hartl, N. Hörmann, J. Hrubec, M. Jeitler, A. König, I. Krätschmer, D. Liko, T. Matsushita, I. Mikulec, D. Rabady, N. Rad, B. Rahbaran, H. Rohringer, J. Schieck, J. Strauss, W. Waltenberger, C.-E. Wulz, O. Dvornikov, V. Makarenko, V. Mossolov, J. Suarez Gonzalez, V. Zykunov, N. Shumeiko, S. Alderweireldt, E. A. De Wolf, X. Janssen, J. Lauwers, M. Van De Klundert, H. Van Haevermaet, P. Van Mechelen, N. Van Remortel, A. Van Spilbeeck, S. Abu Zeid, F. Blekman, J. D’Hondt, N. Daci, I. De Bruyn, K. Deroover, S. Lowette, S. Moortgat, L. Moreels, A. Olbrechts, Q. Python, K. Skovpen, S. Tavernier, W. Van Doninck, P. Van Mulders, I. Van Parijs, H. Brun, B. Clerbaux, G. De Lentdecker, H. Delannoy, G. Fasanella, L. Favart, R. Goldouzian, A. Grebenyuk, G. Karapostoli, T. Lenzi, A. Léonard, J. Luetic, T. Maerschalk, A. Marinov, A. Randle-conde, T. Seva, C. Vander Velde, P. Vanlaer, D. Vannerom, R. Yonamine, F. Zenoni, F. Zhang, A. Cimmino, T. Cornelis, D. Dobur, A. Fagot, M. Gul, I. Khvastunov, D. Poyraz, S. Salva, R. Schöfbeck, M. Tytgat, W. Van Driessche, E. Yazgan, N. Zaganidis, H. Bakhshiansohi, C. Beluffi, O. Bondu, S. Brochet, G. Bruno, A. Caudron, S. De Visscher, C. Delaere, M. Delcourt, B. Francois, A. Giammanco, A. Jafari, M. Komm, G. Krintiras, V. Lemaitre, A. Magitteri, A. Mertens, M. Musich, K. Piotrzkowski, L. Quertenmont, M. Selvaggi, M. Vidal Marono, S. Wertz, N. Beliy, W. L. Aldá Júnior, F. L. Alves, G. A. Alves, L. Brito, C. Hensel, A. Moraes, M. E. Pol, P. Rebello Teles, E. Belchior Batista Das Chagas, W. Carvalho, J. Chinellato, A. Custódio, E. M. Da Costa, G. G. Da Silveira, D. De Jesus Damiao, C. De Oliveira Martins, S. Fonseca De Souza, L. M. Huertas Guativa, H. Malbouisson, D. Matos Figueiredo, C. Mora Herrera, L. Mundim, H. Nogima, W. L. Prado Da Silva, A. Santoro, A. Sznajder, E. J. Tonelli Manganote, F. Torres Da Silva De Araujo, A. Vilela Pereira, S. Ahuja, C. A. Bernardes, S. Dogra, T. R. Fernandez Perez Tomei, E. M. Gregores, P. G. Mercadante, C. S. Moon, S. F. Novaes, Sandra S. Padula, D. Romero Abad, J. C. Ruiz Vargas, A. Aleksandrov, R. Hadjiiska, P. Iaydjiev, M. Rodozov, S. Stoykova, G. Sultanov, M. Vutova, A. Dimitrov, I. Glushkov, L. Litov, B. Pavlov, P. Petkov, W. Fang, M. Ahmad, J. G. Bian, G. M. Chen, H. S. Chen, M. Chen, Y. Chen, T. Cheng, C. H. Jiang, D. Leggat, Z. Liu, F. Romeo, M. Ruan, S. M. Shaheen, A. Spiezia, J. Tao, C. Wang, Z. Wang, H. Zhang, J. Zhao, Y. Ban, G. Chen, Q. Li, S. Liu, Y. Mao, S. J. Qian, D. Wang, Z. Xu, C. Avila, A. Cabrera, L. F. Chaparro Sierra, C. Florez, J. P. Gomez, C. F. González Hernández, J. D. Ruiz Alvarez, J. C. Sanabria, N. Godinovic, D. Lelas, I. Puljak, P. M. Ribeiro Cipriano, T. Sculac, Z. Antunovic, M. Kovac, V. Brigljevic, D. Ferencek, K. Kadija, B. Mesic, T. Susa, A. Attikis, G. Mavromanolakis, J. Mousa, C. Nicolaou, F. Ptochos, P. A. Razis, H. Rykaczewski, D. Tsiakkouri, M. Finger, M. Finger Jr., E. Carrera Jarrin, A. A. Abdelalim, Y. Mohammed, E. Salama, M. Kadastik, L. Perrini, M. Raidal, A. Tiko, C. Veelken, P. Eerola, J. Pekkanen, M. Voutilainen, J. Härkönen, T. Järvinen, V. Karimäki, R. Kinnunen, T. Lampén, K. Lassila-Perini, S. Lehti, T. Lindén, P. Luukka, J. Tuominiemi, E. Tuovinen, L. Wendland, J. Talvitie, T. Tuuva, M. Besancon, F. Couderc, M. Dejardin, D. Denegri, B. Fabbro, J. L. Faure, C. Favaro, F. Ferri, S. Ganjour, S. Ghosh, A. Givernaud, P. Gras, G. Hamel de Monchenault, P. Jarry, I. Kucher, E. Locci, M. Machet, J. Malcles, J. Rander, A. Rosowsky, M. Titov, A. Abdulsalam, I. Antropov, S. Baffioni, F. Beaudette, P. Busson, L. Cadamuro, E. Chapon, C. Charlot, O. Davignon, R. Granier de Cassagnac, M. Jo, S. Lisniak, P. Miné, M. Nguyen, C. Ochando, G. Ortona, P. Paganini, P. Pigard, S. Regnard, R. Salerno, Y. Sirois, A. G. Stahl Leiton, T. Strebler, Y. Yilmaz, A. Zabi, A. Zghiche, J.-L. Agram, J. Andrea, A. Aubin, D. Bloch, J.-M. Brom, M. Buttignol, E. C. Chabert, N. Chanon, C. Collard, E. Conte, X. Coubez, J.-C. Fontaine, D. Gelé, U. Goerlach, A.-C. Le Bihan, P. Van Hove, S. Gadrat, S. Beauceron, C. Bernet, G. Boudoul, C. A. Carrillo Montoya, R. Chierici, D. Contardo, B. Courbon, P. Depasse, H. El Mamouni, J. Fay, S. Gascon, M. Gouzevitch, G. Grenier, B. Ille, F. Lagarde, I. B. Laktineh, M. Lethuillier, L. Mirabito, A. L. Pequegnot, S. Perries, A. Popov, D. Sabes, V. Sordini, M. Vander Donckt, P. Verdier, S. Viret, A. Khvedelidze, Z. Tsamalaidze, C. Autermann, S. Beranek, L. Feld, M. K. Kiesel, K. Klein, M. Lipinski, M. Preuten, C. Schomakers, J. Schulz, T. Verlage, A. Albert, M. Brodski, E. Dietz-Laursonn, D. Duchardt, M. Endres, M. Erdmann, S. Erdweg, T. Esch, R. Fischer, A. Güth, M. Hamer, T. Hebbeker, C. Heidemann, K. Hoepfner, S. Knutzen, M. Merschmeyer, A. Meyer, P. Millet, S. Mukherjee, M. Olschewski, K. Padeken, T. Pook, M. Radziej, H. Reithler, M. Rieger, F. Scheuch, L. Sonnenschein, D. Teyssier, S. Thüer, V. Cherepanov, G. Flügge, B. Kargoll, T. Kress, A. Künsken, J. Lingemann, T. Müller, A. Nehrkorn, A. Nowack, C. Pistone, O. Pooth, A. Stahl, M. Aldaya Martin, T. Arndt, C. Asawatangtrakuldee, K. Beernaert, O. Behnke, U. Behrens, A. A. Bin Anuar, K. Borras, A. Campbell, P. Connor, C. Contreras-Campana, F. Costanza, C. Diez Pardos, G. Dolinska, G. Eckerlin, D. Eckstein, T. Eichhorn, E. Eren, E. Gallo, J. Garay Garcia, A. Geiser, A. Gizhko, J. M. Grados Luyando, A. Grohsjean, P. Gunnellini, A. Harb, J. Hauk, M. Hempel, H. Jung, A. Kalogeropoulos, O. Karacheban, M. Kasemann, J. Keaveney, C. Kleinwort, I. Korol, D. Krücker, W. Lange, A. Lelek, T. Lenz, J. Leonard, K. Lipka, A. Lobanov, W. Lohmann, R. Mankel, I.-A. Melzer-Pellmann, A. B. Meyer, G. Mittag, J. Mnich, A. Mussgiller, D. Pitzl, R. Placakyte, A. Raspereza, B. Roland, M. Ö. Sahin, P. Saxena, T. Schoerner-Sadenius, S. Spannagel, N. Stefaniuk, G. P. Van Onsem, R. Walsh, C. Wissing, V. Blobel, M. Centis Vignali, A. R. Draeger, T. Dreyer, E. Garutti, D. Gonzalez, J. Haller, M. Hoffmann, A. Junkes, R. Klanner, R. Kogler, N. Kovalchuk, T. Lapsien, I. Marchesini, D. Marconi, M. Meyer, M. Niedziela, D. Nowatschin, F. Pantaleo, T. Peiffer, A. Perieanu, C. Scharf, P. Schleper, A. Schmidt, S. Schumann, J. Schwandt, H. Stadie, G. Steinbrück, F. M. Stober, M. Stöver, H. Tholen, D. Troendle, E. Usai, L. Vanelderen, A. Vanhoefer, B. Vormwald, M. Akbiyik, C. Barth, S. Baur, C. Baus, J. Berger, E. Butz, R. Caspart, T. Chwalek, F. Colombo, W. De Boer, A. Dierlamm, S. Fink, B. Freund, R. Friese, M. Giffels, A. Gilbert, P. Goldenzweig, D. Haitz, F. Hartmann, S. M. Heindl, U. Husemann, I. Katkov, S. Kudella, H. Mildner, M. U. Mozer, Th. Müller, M. Plagge, G. Quast, K. Rabbertz, S. Röcker, F. Roscher, M. Schröder, I. Shvetsov, G. Sieber, H. J. Simonis, R. Ulrich, S. Wayand, M. Weber, T. Weiler, S. Williamson, C. Wöhrmann, R. Wolf, G. Anagnostou, G. Daskalakis, T. Geralis, V. A. Giakoumopoulou, A. Kyriakis, D. Loukas, I. Topsis-Giotis, S. Kesisoglou, A. Panagiotou, N. Saoulidou, E. Tziaferi, I. Evangelou, G. Flouris, C. Foudas, P. Kokkas, N. Loukas, N. Manthos, I. Papadopoulos, E. Paradas, N. Filipovic, G. Pasztor, G. Bencze, C. Hajdu, D. Horvath, F. Sikler, V. Veszpremi, G. Vesztergombi, A. J. Zsigmond, N. Beni, S. Czellar, J. Karancsi, A. Makovec, J. Molnar, Z. Szillasi, M. Bartók, P. Raics, Z. L. Trocsanyi, B. Ujvari, J. R. Komaragiri, S. Bahinipati, S. Bhowmik, S. Choudhury, P. Mal, K. Mandal, A. Nayak, D. K. Sahoo, N. Sahoo, S. K. Swain, S. Bansal, S. B. Beri, V. Bhatnagar, R. Chawla, U. Bhawandeep, A. K. Kalsi, A. Kaur, M. Kaur, R. Kumar, P. Kumari, A. Mehta, M. Mittal, J. B. Singh, G. Walia, Ashok Kumar, A. Bhardwaj, B. C. Choudhary, R. B. Garg, S. Keshri, S. Malhotra, M. Naimuddin, K. Ranjan, R. Sharma, V. Sharma, R. Bhattacharya, S. Bhattacharya, K. Chatterjee, S. Dey, S. Dutt, S. Dutta, S. Ghosh, N. Majumdar, A. Modak, K. Mondal, S. Mukhopadhyay, S. Nandan, A. Purohit, A. Roy, D. Roy, S. Roy Chowdhury, S. Sarkar, M. Sharan, S. Thakur, P. K. Behera, R. Chudasama, D. Dutta, V. Jha, V. Kumar, A. K. Mohanty, P. K. Netrakanti, L. M. Pant, P. Shukla, A. Topkar, T. Aziz, S. Dugad, G. Kole, B. Mahakud, S. Mitra, G. B. Mohanty, B. Parida, N. Sur, B. Sutar, S. Banerjee, R. K. Dewanjee, S. Ganguly, M. Guchait, Sa. Jain, S. Kumar, M. Maity, G. Majumder, K. Mazumdar, T. Sarkar, N. Wickramage, S. Chauhan, S. Dube, V. Hegde, A. Kapoor, K. Kothekar, S. Pandey, A. Rane, S. Sharma, S. Chenarani, E. Eskandari Tadavani, S. M. Etesami, M. Khakzad, M. Mohammadi Najafabadi, M. Naseri, S. Paktinat Mehdiabadi, F. Rezaei Hosseinabadi, B. Safarzadeh, M. Zeinali, M. Felcini, M. Grunewald, M. Abbrescia, C. Calabria, C. Caputo, A. Colaleo, D. Creanza, L. Cristella, N. De Filippis, M. De Palma, L. Fiore, G. Iaselli, G. Maggi, M. Maggi, G. Miniello, S. My, S. Nuzzo, A. Pompili, G. Pugliese, R. Radogna, A. Ranieri, G. Selvaggi, A. Sharma, L. Silvestris, R. Venditti, P. Verwilligen, G. Abbiendi, C. Battilana, D. Bonacorsi, S. Braibant-Giacomelli, L. Brigliadori, R. Campanini, P. Capiluppi, A. Castro, F. R. Cavallo, S. S. Chhibra, G. Codispoti, M. Cuffiani, G. M. Dallavalle, F. Fabbri, A. Fanfani, D. Fasanella, P. Giacomelli, C. Grandi, L. Guiducci, S. Marcellini, G. Masetti, A. Montanari, F. L. Navarria, A. Perrotta, A. M. Rossi, T. Rovelli, G. P. Siroli, N. Tosi, S. Albergo, S. Costa, A. Di Mattia, F. Giordano, R. Potenza, A. Tricomi, C. Tuve, G. Barbagli, V. Ciulli, C. Civinini, R. D’Alessandro, E. Focardi, P. Lenzi, M. Meschini, S. Paoletti, L. Russo, G. Sguazzoni, D. Strom, L. Viliani, L. Benussi, S. Bianco, F. Fabbri, D. Piccolo, F. Primavera, V. Calvelli, F. Ferro, M. R. Monge, E. Robutti, S. Tosi, L. Brianza, F. Brivio, V. Ciriolo, M. E. Dinardo, S. Fiorendi, S. Gennai, A. Ghezzi, P. Govoni, M. Malberti, S. Malvezzi, R. A. Manzoni, D. Menasce, L. Moroni, M. Paganoni, D. Pedrini, S. Pigazzini, S. Ragazzi, T. Tabarelli de Fatis, S. Buontempo, N. Cavallo, G. De Nardo, S. Di Guida, M. Esposito, F. Fabozzi, F. Fienga, A. O. M. Iorio, G. Lanza, L. Lista, S. Meola, P. Paolucci, C. Sciacca, F. Thyssen, P. Azzi, N. Bacchetta, L. Benato, D. Bisello, A. Boletti, R. Carlin, A. Carvalho Antunes de Oliveira, P. Checchia, M. Dall’Osso, P. De Castro Manzano, T. Dorigo, U. Dosselli, F. Gasparini, U. Gasparini, A. Gozzelino, S. Lacaprara, M. Margoni, A. T. Meneguzzo, J. Pazzini, N. Pozzobon, P. Ronchese, F. Simonetto, E. Torassa, M. Zanetti, P. Zotto, G. Zumerle, A. Braghieri, F. Fallavollita, A. Magnani, P. Montagna, S. P. Ratti, V. Re, C. Riccardi, P. Salvini, I. Vai, P. Vitulo, L. Alunni Solestizi, G. M. Bilei, D. Ciangottini, L. Fanò, P. Lariccia, R. Leonardi, G. Mantovani, M. Menichelli, A. Saha, A. Santocchia, K. Androsov, P. Azzurri, G. Bagliesi, J. Bernardini, T. Boccali, R. Castaldi, M. A. Ciocci, R. Dell’Orso, S. Donato, G. Fedi, A. Giassi, M. T. Grippo, F. Ligabue, T. Lomtadze, L. Martini, A. Messineo, F. Palla, A. Rizzi, A. Savoy-Navarro, P. Spagnolo, R. Tenchini, G. Tonelli, A. Venturi, P. G. Verdini, L. Barone, F. Cavallari, M. Cipriani, D. Del Re, M. Diemoz, S. Gelli, E. Longo, F. Margaroli, B. Marzocchi, P. Meridiani, G. Organtini, R. Paramatti, F. Preiato, S. Rahatlou, C. Rovelli, F. Santanastasio, N. Amapane, R. Arcidiacono, S. Argiro, M. Arneodo, N. Bartosik, R. Bellan, C. Biino, N. Cartiglia, F. Cenna, M. Costa, R. Covarelli, A. Degano, N. Demaria, L. Finco, B. Kiani, C. Mariotti, S. Maselli, E. Migliore, V. Monaco, E. Monteil, M. Monteno, M. M. Obertino, L. Pacher, N. Pastrone, M. Pelliccioni, G. L. Pinna Angioni, F. Ravera, A. Romero, M. Ruspa, R. Sacchi, K. Shchelina, V. Sola, A. Solano, A. Staiano, P. Traczyk, S. Belforte, M. Casarsa, F. Cossutti, G. Della Ricca, A. Zanetti, D. H. Kim, G. N. Kim, M. S. Kim, S. Lee, S. W. Lee, Y. D. Oh, S. Sekmen, D. C. Son, Y. C. Yang, A. Lee, H. Kim, J. A. Brochero Cifuentes, T. J. Kim, S. Cho, S. Choi, Y. Go, D. Gyun, S. Ha, B. Hong, Y. Jo, Y. Kim, K. Lee, K. S. Lee, S. Lee, J. Lim, S. K. Park, Y. Roh, J. Almond, J. Kim, H. Lee, S. B. Oh, B. C. Radburn-Smith, S. h. Seo, U. K. Yang, H. D. Yoo, G. B. Yu, M. Choi, H. Kim, J. H. Kim, J. S. H. Lee, I. C. Park, G. Ryu, M. S. Ryu, Y. Choi, J. Goh, C. Hwang, J. Lee, I. Yu, V. Dudenas, A. Juodagalvis, J. Vaitkus, I. Ahmed, Z. A. Ibrahim, M. A. B. Md Ali, F. Mohamad Idris, W. A. T. Wan Abdullah, M. N. Yusli, Z. Zolkapli, H. Castilla-Valdez, E. De La Cruz-Burelo, I. Heredia-De La Cruz, A. Hernandez-Almada, R. Lopez-Fernandez, R. Magaña Villalba, J. Mejia Guisao, A. Sanchez-Hernandez, S. Carrillo Moreno, C. Oropeza Barrera, F. Vazquez Valencia, S. Carpinteyro, I. Pedraza, H. A. Salazar Ibarguen, C. Uribe Estrada, A. Morelos Pineda, D. Krofcheck, P. H. Butler, A. Ahmad, M. Ahmad, Q. Hassan, H. R. Hoorani, W. A. Khan, A. Saddique, M. A. Shah, M. Shoaib, M. Waqas, H. Bialkowska, M. Bluj, B. Boimska, T. Frueboes, M. Górski, M. Kazana, K. Nawrocki, K. Romanowska-Rybinska, M. Szleper, P. Zalewski, K. Bunkowski, A. Byszuk, K. Doroba, A. Kalinowski, M. Konecki, J. Krolikowski, M. Misiura, M. Olszewski, M. Walczak, P. Bargassa, C. Beirão Da Cruz E Silva, B. Calpas, A. Di Francesco, P. Faccioli, P. G. Ferreira Parracho, M. Gallinaro, J. Hollar, N. Leonardo, L. Lloret Iglesias, M. V. Nemallapudi, J. Rodrigues Antunes, J. Seixas, O. Toldaiev, D. Vadruccio, J. Varela, S. Afanasiev, P. Bunin, M. Gavrilenko, I. Golutvin, I. Gorbunov, A. Kamenev, V. Karjavin, A. Lanev, A. Malakhov, V. Matveev, V. Palichik, V. Perelygin, S. Shmatov, S. Shulha, N. Skatchkov, V. Smirnov, N. Voytishin, A. Zarubin, L. Chtchipounov, V. Golovtsov, Y. Ivanov, V. Kim, E. Kuznetsova, V. Murzin, V. Oreshkin, V. Sulimov, A. Vorobyev, Yu. Andreev, A. Dermenev, S. Gninenko, N. Golubev, A. Karneyeu, M. Kirsanov, N. Krasnikov, A. Pashenkov, D. Tlisov, A. Toropin, V. Epshteyn, V. Gavrilov, N. Lychkovskaya, V. Popov, I. Pozdnyakov, G. Safronov, A. Spiridonov, M. Toms, E. Vlasov, A. Zhokin, T. Aushev, A. Bylinkin, R. Chistov, S. Polikarpov, E. Zhemchugov, V. Andreev, M. Azarkin, I. Dremin, M. Kirakosyan, A. Leonidov, A. Terkulov, A. Baskakov, A. Belyaev, E. Boos, V. Bunichev, M. Dubinin, L. Dudko, A. Ershov, V. Klyukhin, N. Korneeva, I. Lokhtin, I. Miagkov, S. Obraztsov, M. Perfilov, V. Savrin, P. Volkov, V. Blinov, Y. Skovpen, D. Shtol, I. Azhgirey, I. Bayshev, S. Bitioukov, D. Elumakhov, V. Kachanov, A. Kalinin, D. Konstantinov, V. Krychkine, V. Petrov, R. Ryutin, A. Sobol, S. Troshin, N. Tyurin, A. Uzunian, A. Volkov, P. Adzic, P. Cirkovic, D. Devetak, M. Dordevic, J. Milosevic, V. Rekovic, J. Alcaraz Maestre, M. Barrio Luna, E. Calvo, M. Cerrada, M. Chamizo Llatas, N. Colino, B. De La Cruz, A. Delgado Peris, A. Escalante Del Valle, C. Fernandez, J. P. Fernández Ramos, J. Flix, M. C. Fouz, P. Garcia-Abia, O. Gonzalez Lopez, S. Goy Lopez, J. M. Hernandez, M. I. Josa, E. Navarro De Martino, A. Pérez-Calero Yzquierdo, J. Puerta Pelayo, A. Quintario Olmeda, I. Redondo, L. Romero, M. S. Soares, J. F. de Trocóniz, M. Missiroli, D. Moran, J. Cuevas, J. Fernandez Menendez, I. Gonzalez Caballero, J. R. González Fernández, E. Palencia Cortezon, S. Sanchez Cruz, I. Suárez Andrés, P. Vischia, J. M. Vizan Garcia, I. J. Cabrillo, A. Calderon, E. Curras, M. Fernandez, J. Garcia-Ferrero, G. Gomez, A. Lopez Virto, J. Marco, C. Martinez Rivero, F. Matorras, J. Piedra Gomez, T. Rodrigo, A. Ruiz-Jimeno, L. Scodellaro, N. Trevisani, I. Vila, R. Vilar Cortabitarte, D. Abbaneo, E. Auffray, G. Auzinger, P. Baillon, A. H. Ball, D. Barney, P. Bloch, A. Bocci, C. Botta, T. Camporesi, R. Castello, M. Cepeda, G. Cerminara, Y. Chen, D. d’Enterria, A. Dabrowski, V. Daponte, A. David, M. De Gruttola, A. De Roeck, E. Di Marco, M. Dobson, B. Dorney, T. du Pree, D. Duggan, M. Dünser, N. Dupont, A. Elliott-Peisert, P. Everaerts, S. Fartoukh, G. Franzoni, J. Fulcher, W. Funk, D. Gigi, K. Gill, M. Girone, F. Glege, D. Gulhan, S. Gundacker, M. Guthoff, P. Harris, J. Hegeman, V. Innocente, P. Janot, J. Kieseler, H. Kirschenmann, V. Knünz, A. Kornmayer, M. J. Kortelainen, K. Kousouris, M. Krammer, C. Lange, P. Lecoq, C. Lourenço, M. T. Lucchini, L. Malgeri, M. Mannelli, A. Martelli, F. Meijers, J. A. Merlin, S. Mersi, E. Meschi, P. Milenovic, F. Moortgat, S. Morovic, M. Mulders, H. Neugebauer, S. Orfanelli, L. Orsini, L. Pape, E. Perez, M. Peruzzi, A. Petrilli, G. Petrucciani, A. Pfeiffer, M. Pierini, A. Racz, T. Reis, G. Rolandi, M. Rovere, H. Sakulin, J. B. Sauvan, C. Schäfer, C. Schwick, M. Seidel, A. Sharma, P. Silva, P. Sphicas, J. Steggemann, M. Stoye, Y. Takahashi, M. Tosi, D. Treille, A. Triossi, A. Tsirou, V. Veckalns, G. I. Veres, M. Verweij, N. Wardle, H. K. Wöhri, A. Zagozdzinska, W. D. Zeuner, W. Bertl, K. Deiters, W. Erdmann, R. Horisberger, Q. Ingram, H. C. Kaestli, D. Kotlinski, U. Langenegger, T. Rohe, S. A. Wiederkehr, F. Bachmair, L. Bäni, L. Bianchini, B. Casal, G. Dissertori, M. Dittmar, M. Donegà, C. Grab, C. Heidegger, D. Hits, J. Hoss, G. Kasieczka, W. Lustermann, B. Mangano, M. Marionneau, P. Martinez Ruiz del Arbol, M. Masciovecchio, M. T. Meinhard, D. Meister, F. Micheli, P. Musella, F. Nessi-Tedaldi, F. Pandolfi, J. Pata, F. Pauss, G. Perrin, L. Perrozzi, M. Quittnat, M. Rossini, M. Schönenberger, A. Starodumov, V. R. Tavolaro, K. Theofilatos, R. Wallny, T. K. Aarrestad, C. Amsler, L. Caminada, M. F. Canelli, A. De Cosa, C. Galloni, A. Hinzmann, T. Hreus, B. Kilminster, J. Ngadiuba, D. Pinna, G. Rauco, P. Robmann, D. Salerno, C. Seitz, Y. Yang, A. Zucchetta, V. Candelise, T. H. Doan, Sh. Jain, R. Khurana, M. Konyushikhin, C. M. Kuo, W. Lin, A. Pozdnyakov, S. S. Yu, Arun Kumar, P. Chang, Y. H. Chang, Y. Chao, K. F. Chen, P. H. Chen, F. Fiori, W. -S. Hou, Y. Hsiung, Y. F. Liu, R. -S. Lu, M. Miñano Moya, E. Paganis, A. Psallidas, J. f. Tsai, B. Asavapibhop, G. Singh, N. Sri manobhas, N. Suwonjandee, A. Adiguzel, M. N. Bakirci, S. Damarseckin, Z. S. Demiroglu, C. Dozen, E. Eskut, S. Girgis, G. Gokbulut, Y. Guler, I. Hos, E. E. Kangal, O. Kara, U. Kiminsu, M. Oglakci, G. Onengut, K. Ozdemir, S. Ozturk, A. Polatoz, D. Sunar Cerci, S. Turkcapar, I. S. Zorbakir, C. Zorbilmez, B. Bilin, S. Bilmis, B. Isildak, G. Karapinar, M. Yalvac, M. Zeyrek, E. Gülmez, M. Kaya, O. Kaya, E. A. Yetkin, T. Yetkin, A. Cakir, K. Cankocak, S. Sen, B. Grynyov, L. Levchuk, P. Sorokin, R. Aggleton, F. Ball, L. Beck, J. J. Brooke, D. Burns, E. Clement, D. Cussans, H. Flacher, J. Goldstein, M. Grimes, G. P. Heath, H. F. Heath, J. Jacob, L. Kreczko, C. Lucas, D. M. Newbold, S. Paramesvaran, A. Poll, T. Sakuma, S. Seif El Nasr-storey, D. Smith, V. J. Smith, K. W. Bell, A. Belyaev, C. Brew, R. M. Brown, L. Calligaris, D. Cieri, D. J. A. Cockerill, J. A. Coughlan, K. Harder, S. Harper, E. Olaiya, D. Petyt, C. H. Shepherd-Themistocleous, A. Thea, I. R. Tomalin, T. Williams, M. Baber, R. Bainbridge, O. Buchmuller, A. Bundock, D. Burton, S. Casasso, M. Citron, D. Colling, L. Corpe, P. Dauncey, G. Davies, A. De Wit, M. Della Negra, R. Di Maria, P. Dunne, A. Elwood, D. Futyan, Y. Haddad, G. Hall, G. Iles, T. James, R. Lane, C. Laner, R. Lucas, L. Lyons, A.-M. Magnan, S. Malik, L. Mastrolorenzo, J. Nash, A. Nikitenko, J. Pela, B. Penning, M. Pesaresi, D. M. Raymond, A. Richards, A. Rose, E. Scott, C. Seez, S. Summers, A. Tapper, K. Uchida, M. Vazquez Acosta, T. Virdee, J. Wright, S. C. Zenz, J. E. Cole, P. R. Hobson, A. Khan, P. Kyberd, I. D. Reid, P. Symonds, L. Teodorescu, M. Turner, A. Borzou, K. Call, J. Dittmann, K. Hatakeyama, H. Liu, N. Pastika, R. Bartek, A. Dominguez, A. Buccilli, S. I. Cooper, C. Henderson, P. Rumerio, C. West, D. Arcaro, A. Avetisyan, T. Bose, D. Gastler, D. Rankin, C. Richardson, J. Rohlf, L. Sulak, D. Zou, G. Benelli, D. Cutts, A. Garabedian, J. Hakala, U. Heintz, J. M. Hogan, O. Jesus, K. H. M. Kwok, E. Laird, G. Landsberg, Z. Mao, M. Narain, S. Piperov, S. Sagir, E. Spencer, R. Syarif, R. Breedon, D. Burns, M. Calderon De La Barca Sanchez, S. Chauhan, M. Chertok, J. Conway, R. Conway, P. T. Cox, R. Erbacher, C. Flores, G. Funk, M. Gardner, W. Ko, R. Lander, C. Mclean, M. Mulhearn, D. Pellett, J. Pilot, S. Shalhout, M. Shi, J. Smith, M. Squires, D. Stolp, K. Tos, M. Tripathi, M. Bachtis, C. Bravo, R. Cousins, A. Dasgupta, A. Florent, J. Hauser, M. Ignatenko, N. Mccoll, D. Saltzberg, C. Schnaible, V. Valuev, M. Weber, E. Bouvier, K. Burt, R. Clare, J. Ellison, J. W. Gary, S. M. A. Ghiasi Shirazi, G. Hanson, J. Heilman, P. Jandir, E. Kennedy, F. Lacroix, O. R. Long, M. Olmedo Negrete, M. I. Paneva, A. Shrinivas, W. Si, H. Wei, S. Wimpenny, B. R. Yates, J. G. Branson, G. B. Cerati, S. Cittolin, M. Derdzinski, R. Gerosa, A. Holzner, D. Klein, V. Krutelyov, J. Letts, I. Macneill, D. Olivito, S. Padhi, M. Pieri, M. Sani, V. Sharma, S. Simon, M. Tadel, A. Vartak, S. Wasserbaech, C. Welke, J. Wood, F. Würthwein, A. Yagil, G. Zevi Della Porta, N. Amin, R. Bhandari, J. Bradmiller-Feld, C. Campagnari, A. Dishaw, V. Dutta, M. Franco Sevilla, C. George, F. Golf, L. Gouskos, J. Gran, R. Heller, J. Incandela, S. D. Mullin, A. Ovcharova, H. Qu, J. Richman, D. Stuart, I. Suarez, J. Yoo, D. Anderson, J. Bendavid, A. Bornheim, J. Bunn, J. Duarte, J. M. Lawhorn, A. Mott, H. B. Newman, C. Pena, M. Spiropulu, J. R. Vlimant, S. Xie, R. Y. Zhu, M. B. Andrews, T. Ferguson, M. Paulini, J. Russ, M. Sun, H. Vogel, I. Vorobiev, M. Weinberg, J. P. Cumalat, W. T. Ford, F. Jensen, A. Johnson, M. Krohn, S. Leontsinis, T. Mulholland, K. Stenson, S. R. Wagner, J. Alexander, J. Chaves, J. Chu, S. Dittmer, K. Mcdermott, N. Mirman, G. Nicolas Kaufman, J. R. Patterson, A. Rinkevicius, A. Ryd, L. Skinnari, L. Soffi, S. M. Tan, Z. Tao, J. Thom, J. Tucker, P. Wittich, M. Zientek, D. Winn, S. Abdullin, M. Albrow, G. Apollinari, A. Apresyan, S. Banerjee, L. A. T. Bauerdick, A. Beretvas, J. Berryhill, P. C. Bhat, G. Bolla, K. Burkett, J. N. Butler, H. W. K. Cheung, F. Chlebana, S. Cihangir, M. Cremonesi, V. D. Elvira, I. Fisk, J. Freeman, E. Gottschalk, L. Gray, D. Green, S. Grünendahl, O. Gutsche, D. Hare, R. M. Harris, S. Hasegawa, J. Hirschauer, Z. Hu, B. Jayatilaka, S. Jindariani, M. Johnson, U. Joshi, B. Klima, B. Kreis, S. Lammel, J. Linacre, D. Lincoln, R. Lipton, M. Liu, T. Liu, R. Lopes De Sá, J. Lykken, K. Maeshima, N. Magini, J. M. Marraffino, S. Maruyama, D. Mason, P. McBride, P. Merkel, S. Mrenna, S. Nahn, V. O’Dell, K. Pedro, O. Prokofyev, G. Rakness, L. Ristori, E. Sexton-Kennedy, A. Soha, W. J. Spalding, L. Spiegel, S. Stoynev, J. Strait, N. Strobbe, L. Taylor, S. Tkaczyk, N. V. Tran, L. Uplegger, E. W. Vaandering, C. Vernieri, M. Verzocchi, R. Vidal, M. Wang, H. A. Weber, A. Whitbeck, Y. Wu, D. Acosta, P. Avery, P. Bortignon, D. Bourilkov, A. Brinkerhoff, A. Carnes, M. Carver, D. Curry, S. Das, R. D. Field, I. K. Furic, J. Konigsberg, A. Korytov, J. F. Low, P. Ma, K. Matchev, H. Mei, G. Mitselmakher, D. Rank, L. Shchutska, D. Sperka, L. Thomas, J. Wang, S. Wang, J. Yelton, S. Linn, P. Markowitz, G. Martinez, J. L. Rodriguez, A. Ackert, T. Adams, A. Askew, S. Bein, S. Hagopian, V. Hagopian, K. F. Johnson, T. Kolberg, H. Prosper, A. Santra, R. Yohay, M. M. Baarmand, V. Bhopatkar, S. Colafranceschi, M. Hohlmann, D. Noonan, T. Roy, F. Yumiceva, M. R. Adams, L. Apanasevich, D. Berry, R. R. Betts, I. Bucinskaite, R. Cavanaugh, O. Evdokimov, L. Gauthier, C. E. Gerber, D. J. Hofman, K. Jung, I. D. Sandoval Gonzalez, N. Varelas, H. Wang, Z. Wu, M. Zakaria, J. Zhang, B. Bilki, W. Clarida, K. Dilsiz, S. Durgut, R. P. Gandrajula, M. Haytmyradov, V. Khristenko, J.-P. Merlo, H. Mermerkaya, A. Mestvirishvili, A. Moeller, J. Nachtman, H. Ogul, Y. Onel, F. Ozok, A. Penzo, C. Snyder, E. Tiras, J. Wetzel, K. Yi, B. Blumenfeld, A. Cocoros, N. Eminizer, D. Fehling, L. Feng, A. V. Gritsan, P. Maksimovic, J. Roskes, U. Sarica, M. Swartz, M. Xiao, C. You, A. Al-bataineh, P. Baringer, A. Bean, S. Boren, J. Bowen, J. Castle, L. Forthomme, R. P. Kenny III, S. Khalil, A. Kropivnitskaya, D. Majumder, W. Mcbrayer, M. Murray, S. Sanders, R. Stringer, J. D. Tapia Takaki, Q. Wang, A. Ivanov, K. Kaadze, Y. Maravin, A. Mohammadi, L. K. Saini, N. Skhirtladze, S. Toda, F. Rebassoo, D. Wright, C. Anelli, A. Baden, O. Baron, A. Belloni, B. Calvert, S. C. Eno, C. Ferraioli, J. A. Gomez, N. J. Hadley, S. Jabeen, G. Y. Jeng, R. G. Kellogg, J. Kunkle, A. C. Mignerey, F. Ricci-Tam, Y. H. Shin, A. Skuja, M. B. Tonjes, S. C. Tonwar, D. Abercrombie, B. Allen, A. Apyan, V. Azzolini, R. Barbieri, A. Baty, R. Bi, K. Bierwagen, S. Brandt, W. Busza, I. A. Cali, M. D’Alfonso, Z. Demiragli, G. Gomez Ceballos, M. Goncharov, D. Hsu, Y. Iiyama, G. M. Innocenti, M. Klute, D. Kovalskyi, K. Krajczar, Y. S. Lai, Y.-J. Lee, A. Levin, P. D. Luckey, B. Maier, A. C. Marini, C. Mcginn, C. Mironov, S. Narayanan, X. Niu, C. Paus, C. Roland, G. Roland, J. Salfeld-Nebgen, G. S. F. Stephans, K. Tatar, D. Velicanu, J. Wang, T. W. Wang, B. Wyslouch, A. C. Benvenuti, R. M. Chatterjee, A. Evans, P. Hansen, S. Kalafut, S. C. Kao, Y. Kubota, Z. Lesko, J. Mans, S. Nourbakhsh, N. Ruckstuhl, R. Rusack, N. Tambe, J. Turkewitz, J. G. Acosta, S. Oliveros, E. Avdeeva, K. Bloom, D. R. Claes, C. Fangmeier, R. Gonzalez Suarez, R. Kamalieddin, I. Kravchenko, A. Malta Rodrigues, J. Monroy, J. E. Siado, G. R. Snow, B. Stieger, M. Alyari, J. Dolen, A. Godshalk, C. Harrington, I. Iashvili, J. Kaisen, D. Nguyen, A. Parker, S. Rappoccio, B. Roozbahani, G. Alverson, E. Barberis, A. Hortiangtham, A. Massironi, D. M. Morse, D. Nash, T. Orimoto, R. Teixeira De Lima, D. Trocino, R.-J. Wang, D. Wood, S. Bhattacharya, O. Charaf, K. A. Hahn, A. Kumar, N. Mucia, N. Odell, B. Pollack, M. H. Schmitt, K. Sung, M. Trovato, M. Velasco, N. Dev, M. Hildreth, K. Hurtado Anampa, C. Jessop, D. J. Karmgard, N. Kellams, K. Lannon, N. Marinelli, F. Meng, C. Mueller, Y. Musienko, M. Planer, A. Reinsvold, R. Ruchti, N. Rupprecht, G. Smith, S. Taroni, M. Wayne, M. Wolf, A. Woodard, J. Alimena, L. Antonelli, B. Bylsma, L. S. Durkin, S. Flowers, B. Francis, A. Hart, C. Hill, R. Hughes, W. Ji, B. Liu, W. Luo, D. Puigh, B. L. Winer, H. W. Wulsin, S. Cooperstein, O. Driga, P. Elmer, J. Hardenbrook, P. Hebda, D. Lange, J. Luo, D. Marlow, T. Medvedeva, K. Mei, I. Ojalvo, J. Olsen, C. Palmer, P. Piroué, D. Stickland, A. Svyatkovskiy, C. Tully, S. Malik, A. Barker, V. E. Barnes, S. Folgueras, L. Gutay, M. K. Jha, M. Jones, A. W. Jung, A. Khatiwada, D. H. Miller, N. Neumeister, J. F. Schulte, X. Shi, J. Sun, F. Wang, W. Xie, N. Parashar, J. Stupak, A. Adair, B. Akgun, Z. Chen, K. M. Ecklund, F. J. M. Geurts, M. Guilbaud, W. Li, B. Michlin, M. Northup, B. P. Padley, J. Roberts, J. Rorie, Z. Tu, J. Zabel, B. Betchart, A. Bodek, P. de Barbaro, R. Demina, Y. t. Duh, T. Ferbel, M. Galanti, A. Garcia-Bellido, J. Han, O. Hindrichs, A. Khukhunaishvili, K. H. Lo, P. Tan, M. Verzetti, A. Agapitos, J. P. Chou, Y. Gershtein, T. A. Gómez Espinosa, E. Halkiadakis, M. Heindl, E. Hughes, S. Kaplan, R. Kunnawalkam Elayavalli, S. Kyriacou, A. Lath, K. Nash, M. Osherson, H. Saka, S. Salur, S. Schnetzer, D. Sheffield, S. Somalwar, R. Stone, S. Thomas, P. Thomassen, M. Walker, A. G. Delannoy, M. Foerster, J. Heideman, G. Riley, K. Rose, S. Spanier, K. Thapa, O. Bouhali, A. Celik, M. Dalchenko, M. De Mattia, A. Delgado, S. Dildick, R. Eusebi, J. Gilmore, T. Huang, E. Juska, T. Kamon, R. Mueller, Y. Pakhotin, R. Patel, A. Perloff, L. Perniè, D. Rathjens, A. Safonov, A. Tatarinov, K. A. Ulmer, N. Akchurin, C. Cowden, J. Damgov, F. De Guio, C. Dragoiu, P. R. Dudero, J. Faulkner, E. Gurpinar, S. Kunori, K. Lamichhane, S. W. Lee, T. Libeiro, T. Peltola, S. Undleeb, I. Volobouev, Z. Wang, S. Greene, A. Gurrola, R. Janjam, W. Johns, C. Maguire, A. Melo, H. Ni, P. Sheldon, S. Tuo, J. Velkovska, Q. Xu, M. W. Arenton, P. Barria, B. Cox, J. Goodell, R. Hirosky, A. Ledovskoy, H. Li, C. Neu, T. Sinthuprasith, X. Sun, Y. Wang, E. Wolfe, F. Xia, C. Clarke, R. Harr, P. E. Karchin, J. Sturdy, D. A. Belknap, J. Buchanan, C. Caillol, S. Dasu, L. Dodd, S. Duric, B. Gomber, M. Grothe, M. Herndon, A. Hervé, P. Klabbers, A. Lanaro, A. Levine, K. Long, R. Loveless, T. Perry, G. A. Pierro, G. Polese, T. Ruggles, A. Savin, N. Smith, W. H. Smith, D. Taylor, N. Woods, CMS Collaboration

**Affiliations:** 1 Yerevan Physics Institute, Yerevan, Armenia; 2 Institut für Hochenergiephysik, Wien, Austria; 3 Institute for Nuclear Problems, Minsk, Belarus; 4 National Centre for Particle and High Energy Physics, Minsk, Belarus; 5 Universiteit Antwerpen, Antwerpen, Belgium; 6 Vrije Universiteit Brussel, Brussel, Belgium; 7 Université Libre de Bruxelles, Bruxelles, Belgium; 8 Ghent University, Ghent, Belgium; 9 Université Catholique de Louvain, Louvain-la-Neuve, Belgium; 10 Université de Mons, Mons, Belgium; 11 Centro Brasileiro de Pesquisas Fisicas, Rio de Janeiro, Brazil; 12 Universidade do Estado do Rio de Janeiro, Rio de Janeiro, Brazil; 13 Universidade Estadual Paulista , Universidade Federal do ABC, São Paulo, Brazil; 14 Institute for Nuclear Research and Nuclear Energy, Sofia, Bulgaria; 15 University of Sofia, Sofia, Bulgaria; 16 Beihang University, Beijing, China; 17 Institute of High Energy Physics, Beijing, China; 18 State Key Laboratory of Nuclear Physics and Technology, Peking University, Beijing, China; 19 Universidad de Los Andes, Bogota, Colombia; 20 University of Split, Faculty of Electrical Engineering, Mechanical Engineering and Naval Architecture, Split, Croatia; 21 University of Split, Faculty of Science, Split, Croatia; 22 Institute Rudjer Boskovic, Zagreb, Croatia; 23 University of Cyprus, Nicosia, Cyprus; 24 Charles University, Prague, Czech Republic; 25 Universidad San Francisco de Quito, Quito, Ecuador; 26 Academy of Scientific Research and Technology of the Arab Republic of Egypt, Egyptian Network of High Energy Physics, Cairo, Egypt; 27 National Institute of Chemical Physics and Biophysics, Tallinn, Estonia; 28 Department of Physics, University of Helsinki, Helsinki, Finland; 29 Helsinki Institute of Physics, Helsinki, Finland; 30 Lappeenranta University of Technology, Lappeenranta, Finland; 31 IRFU, CEA, Université Paris-Saclay, Gif-sur-Yvette, France; 32 Laboratoire Leprince-Ringuet, Ecole Polytechnique, IN2P3-CNRS, Palaiseau, France; 33 Institut Pluridisciplinaire Hubert Curien (IPHC), Université de Strasbourg, CNRS-IN2P3, Strasbourg, France; 34 Centre de Calcul de l’Institut National de Physique Nucleaire et de Physique des Particules CNRS/IN2P3, Villeurbanne, France; 35 Université de Lyon, Université Claude Bernard Lyon 1, CNRS-IN2P3, Institut de Physique Nucléaire de Lyon, Villeurbanne, France; 36 Georgian Technical University, Tbilisi, Georgia; 37 Tbilisi State University, Tbilisi, Georgia; 38 RWTH Aachen University, I. Physikalisches Institut, Aachen, Germany; 39 RWTH Aachen University, III. Physikalisches Institut A, Aachen, Germany; 40 RWTH Aachen University, III. Physikalisches Institut B, Aachen, Germany; 41 Deutsches Elektronen-Synchrotron, Hamburg, Germany; 42 University of Hamburg, Hamburg, Germany; 43 Institut für Experimentelle Kernphysik, Karlsruhe, Germany; 44 Institute of Nuclear and Particle Physics (INPP), NCSR Demokritos, Aghia Paraskevi, Greece; 45 National and Kapodistrian University of Athens, Athens, Greece; 46 University of Ioánnina, Ioánnina, Greece; 47 MTA-ELTE Lendület CMS Particle and Nuclear Physics Group, Eötvös Loránd University, Budapest, Hungary; 48 Wigner Research Centre for Physics, Budapest, Hungary; 49 Institute of Nuclear Research ATOMKI, Debrecen, Hungary; 50 Institute of Physics, University of Debrecen, Debrecen, Hungary; 51 Indian Institute of Science (IISc), Bangalore, India; 52 National Institute of Science Education and Research, Bhubaneswar, India; 53 Panjab University, Chandigarh, India; 54 University of Delhi, Delhi, India; 55 Saha Institute of Nuclear Physics, Kolkata, India; 56 Indian Institute of Technology Madras, Madras, India; 57 Bhabha Atomic Research Centre, Mumbai, India; 58 Tata Institute of Fundamental Research-A, Mumbai, India; 59 Tata Institute of Fundamental Research-B, Mumbai, India; 60 Indian Institute of Science Education and Research (IISER), Pune, India; 61 Institute for Research in Fundamental Sciences (IPM), Tehran, Iran; 62 University College Dublin, Dublin, Ireland; 63 INFN Sezione di Bari , Università di Bari , Politecnico di Bari, Bari, Italy; 64 INFN Sezione di Bologna , Università di Bologna, Bologna, Italy; 65 INFN Sezione di Catania , Università di Catania, Catania, Italy; 66 INFN Sezione di Firenze , Università di Firenze, Firenze, Italy; 67 INFN Laboratori Nazionali di Frascati, Frascati, Italy; 68 INFN Sezione di Genova , Università di Genova, Genoa, Italy; 69 INFN Sezione di Milano-Bicocca , Università di Milano-Bicocca, Milan, Italy; 70 INFN Sezione di Napoli , Università di Napoli ’Federico II’ , Naples, Italy, Università della Basilicata , Potenza, Italy, Università G. Marconi, Rome, Italy; 71 INFN Sezione di Padova , Università di Padova , Padua, Italy, Università di Trento, Trento, Italy; 72 INFN Sezione di Pavia , Università di Pavia, Pavia, Italy; 73 INFN Sezione di Perugia , Università di Perugia, Perugia, Italy; 74 INFN Sezione di Pisa , Università di Pisa , Scuola Normale Superiore di Pisa, Pisa, Italy; 75 INFN Sezione di Roma , Università di Roma, Rome, Italy; 76 INFN Sezione di Torino , Università di Torino , Turin, Italy, Università del Piemonte Orientale, Novara, Italy; 77 INFN Sezione di Trieste , Università di Trieste, Trieste, Italy; 78 Kyungpook National University, Taegu, Korea; 79 Chonbuk National University, Chonju, Korea; 80 Chonnam National University Institute for Universe and Elementary Particles, Kwangju, Korea; 81 Hanyang University, Seoul, Korea; 82 Korea University, Seoul, Korea; 83 Seoul National University, Seoul, Korea; 84 University of Seoul, Seoul, Korea; 85 Sungkyunkwan University, Suwon, Korea; 86 Vilnius University, Vilnius, Lithuania; 87 National Centre for Particle Physics, Universiti Malaya, Kuala Lumpur, Malaysia; 88 Centro de Investigacion y de Estudios Avanzados del IPN, Mexico City, Mexico; 89 Universidad Iberoamericana, Mexico City, Mexico; 90 Benemerita Universidad Autonoma de Puebla, Puebla, Mexico; 91 Universidad Autónoma de San Luis Potosí, San Luis Potosí, Mexico; 92 University of Auckland, Auckland, New Zealand; 93 University of Canterbury, Christchurch, New Zealand; 94 National Centre for Physics, Quaid-I-Azam University, Islamabad, Pakistan; 95 National Centre for Nuclear Research, Swierk, Poland; 96 Institute of Experimental Physics Faculty of Physics University of Warsaw, Warsaw, Poland; 97 Laboratório de Instrumentação e Física Experimental de Partículas, Lisbon, Portugal; 98 Joint Institute for Nuclear Research, Dubna, Russia; 99 Petersburg Nuclear Physics Institute, Gatchina (St. Petersburg), Russia; 100 Institute for Nuclear Research, Moscow, Russia; 101 Institute for Theoretical and Experimental Physics, Moscow, Russia; 102 Moscow Institute of Physics and Technology, Moscow, Russia; 103 National Research Nuclear University ’Moscow Engineering Physics Institute’ (MEPhI), Moscow, Russia; 104 P.N. Lebedev Physical Institute, Moscow, Russia; 105 Skobeltsyn Institute of Nuclear Physics Lomonosov Moscow State University, Moscow, Russia; 106 Novosibirsk State University (NSU), Novosibirsk, Russia; 107 State Research Center of Russian Federation Institute for High Energy Physics, Protvino, Russia; 108 University of Belgrade Faculty of Physics and Vinca Institute of Nuclear Sciences, Belgrade, Serbia; 109 Centro de Investigaciones Energéticas Medioambientales y Tecnológicas (CIEMAT), Madrid, Spain; 110 Universidad Autónoma de Madrid, Madrid, Spain; 111 Universidad de Oviedo, Oviedo, Spain; 112 Instituto de Física de Cantabria (IFCA), CSIC-Universidad de Cantabria, Santander, Spain; 113 CERN, European Organization for Nuclear Research, Geneva, Switzerland; 114 Paul Scherrer Institut, Villigen, Switzerland; 115 Institute for Particle Physics ETH Zurich, Zurich, Switzerland; 116 Universität Zürich, Zurich, Switzerland; 117 National Central University, Chung-Li, Taiwan; 118 National Taiwan University (NTU), Taipei, Taiwan; 119 Chulalongkorn University Faculty of Science Department of Physics, Bangkok, Thailand; 120 Physics Department Science and Art Faculty, Cukurova University, Adana, Turkey; 121 Middle East Technical University Physics Department, Ankara, Turkey; 122 Bogazici University, Istanbul, Turkey; 123 Istanbul Technical University, Istanbul, Turkey; 124 Institute for Scintillation Materials of National Academy of Science of Ukraine, Kharkov, Ukraine; 125 National Scientific Center, Kharkov Institute of Physics and Technology, Kharkov, Ukraine; 126 University of Bristol, Bristol, UK; 127 Rutherford Appleton Laboratory, Didcot, UK; 128 Imperial College, London, UK; 129 Brunel University, Uxbridge, UK; 130 Baylor University, Waco, USA; 131 Catholic University of America, Washington, USA; 132 The University of Alabama, Tuscaloosa, USA; 133 Boston University, Boston, USA; 134 Brown University, Providence, USA; 135 University of California Davis, Davis, USA; 136 University of California, Los Angeles, USA; 137 University of California Riverside, Riverside, USA; 138 University of California San Diego, La Jolla, USA; 139 University of California Santa Barbara - Department of Physics, Santa Barbara, USA; 140 California Institute of Technology, Pasadena, USA; 141 Carnegie Mellon University, Pittsburgh, USA; 142 University of Colorado Boulder, Boulder, USA; 143 Cornell University, Ithaca, USA; 144 Fairfield University, Fairfield, USA; 145 Fermi National Accelerator Laboratory, Batavia, USA; 146 University of Florida, Gainesville, USA; 147 Florida International University, Miami, USA; 148 Florida State University, Tallahassee, USA; 149 Florida Institute of Technology, Melbourne, USA; 150 University of Illinois at Chicago (UIC), Chicago, USA; 151 The University of Iowa, Iowa City, USA; 152 Johns Hopkins University, Baltimore, USA; 153 The University of Kansas, Lawrence, USA; 154 Kansas State University, Manhattan, USA; 155 Lawrence Livermore National Laboratory, Livermore, USA; 156 University of Maryland, College Park, USA; 157 Massachusetts Institute of Technology, Cambridge, USA; 158 University of Minnesota, Minneapolis, USA; 159 University of Mississippi, Oxford, USA; 160 University of Nebraska-Lincoln, Lincoln, USA; 161 State University of New York at Buffalo, Buffalo, USA; 162 Northeastern University, Boston, USA; 163 Northwestern University, Evanston, USA; 164 University of Notre Dame, Notre Dame, USA; 165 The Ohio State University, Columbus, USA; 166 Princeton University, Princeton, USA; 167 University of Puerto Rico, Mayaguez, USA; 168 Purdue University, West Lafayette, USA; 169 Purdue University Northwest, Hammond, USA; 170 Rice University, Houston, USA; 171 University of Rochester, Rochester, USA; 172 Rutgers, The State University of New Jersey, Piscataway, USA; 173 University of Tennessee, Knoxville, USA; 174 Texas A&M University, College Station, USA; 175 Texas Tech University, Lubbock, USA; 176 Vanderbilt University, Nashville, USA; 177 University of Virginia, Charlottesville, USA; 178 Wayne State University, Detroit, USA; 179 University of Wisconsin-Madison, Madison, WI, USA; 180 CERN, 1211 Geneva 23, Switzerland

**Keywords:** CMS, Physics, Top quark cross section, Top quark mass

## Abstract

The first measurement of the jet mass $m_{\text {jet}}$ of top quark jets produced in ${\mathrm{t}}\overline{\mathrm{t}}$ events from pp collisions at $\sqrt{s}=8$ $\,\text {TeV}$ is reported for the jet with the largest transverse momentum $p_{\mathrm{T}}$ in highly boosted hadronic top quark decays. The data sample, collected with the CMS detector, corresponds to an integrated luminosity of 19.7$\,\text {fb}^{-1}$. The measurement is performed in the lepton+jets channel in which the products of the semileptonic decay ${\mathrm{t}} \rightarrow \mathrm{b} \mathrm {W}$ with $\mathrm {W}\rightarrow \ell \nu$ where $\ell$ is an electron or muon, are used to select ${\mathrm{t}}\overline{\mathrm{t}}$ events with large Lorentz boosts. The products of the fully hadronic decay ${\mathrm{t}} \rightarrow \mathrm{b} \mathrm {W}$ with $\mathrm {W}\rightarrow \mathrm{q} \overline{\mathrm{q}} '$ are reconstructed using a single Cambridge–Aachen jet with distance parameter $R=1.2$, and $p_{\mathrm{T}} >400$ $\,\text {GeV}$. The ${\mathrm{t}}\overline{\mathrm{t}}$ cross section as a function of $m_{\text {jet}}$ is unfolded at the particle level and is used to test the modelling of highly boosted top quark production. The peak position of the $m_{\text {jet}}$ distribution is sensitive to the top quark mass $m_{{\mathrm{t}}}$, and the data are used to extract a value of $m_{{\mathrm{t}}}$ to assess this sensitivity.

## Introduction

The top quark may play a special role in the standard model (SM) of particle physics owing to its large mass and its possible importance in electroweak symmetry breaking [1, 2]. Measurements of ${\mathrm{t}}\overline{\mathrm{t}}$ production provide crucial information about the accuracy of the SM near the electroweak scale [3, 4], and in assessing the predictions of quantum chromodynamics (QCD) at large mass scales. In turn, they can be used to determine the fundamental parameters of the theory, such as the strong coupling constant or the top quark mass [5, 6].

Previous differential measurements of the ${\mathrm{t}}\overline{\mathrm{t}}$ production cross section [7–15] at the Fermilab Tevatron and CERN LHC show excellent agreement with SM predictions. However, investigations of top quarks with very large transverse momenta $p_{\mathrm{T}}$ have proven to be difficult, since in this kinematic range the decays of the top quark to fully hadronic final states become highly collimated and merge into single jets. In this highly boosted regime, the ${\mathrm{t}}\overline{\mathrm{t}}$ reconstruction efficiency deteriorates for previous, more-traditional measurements. Special reconstruction techniques based on jet substructure are often used to improve the measurements [16, 17] or to implement searches for new physics [18–28]. A detailed understanding of jet substructure observables, and especially the jet mass $m_{\text {jet}}$, is crucial for LHC analyses of highly boosted topologies. While measurements of $m_{\text {jet}}$ corrected to the particle level have been carried out for light-quark and gluon jets [29, 30], the $m_{\text {jet}}$ distribution for highly boosted top quarks has not yet been measured.

Apart from testing the simulation of $m_{\text {jet}}$ in fully hadronic top quark decays, the location of the peak of the $m_{\text {jet}}$ distribution is sensitive to the top quark mass $m_{{\mathrm{t}}}$  [31]. This measurement therefore provides an alternative method of determining $m_{{\mathrm{t}}}$ in the boosted regime, independent of previous mass measurements [32–37]. Calculations from first principles have been performed in soft collinear effective theory [38–41] for the dijet invariant mass distribution from highly boosted top quark production in $\mathrm {e}^+\mathrm {e}^-$ collisions [42, 43], and work is ongoing to extend this to the LHC environment [44, 45]. Such calculations account for perturbative and nonperturbative effects, and provide particle-level predictions. Once predictions for the LHC become available, the measurement of the $m_{\text {jet}}$ distribution can lead to an extraction of $m_{{\mathrm{t}}}$ without the ambiguities that arise from the unknown relation between $m_{{\mathrm{t}}}$ in a well-defined renormalisation scheme and the top quark mass parameter used in Monte Carlo (MC) simulations [45–48].

We present the first measurement of the differential ${\mathrm{t}}\overline{\mathrm{t}}$ production cross section as a function of the leading-jet mass, where leading refers to the jet with the highest $p_{\mathrm{T}}$. The measurement is based on data from $\mathrm {p}\mathrm {p}$ collisions at $\sqrt{s} = 8\,\text {TeV}$, recorded by the CMS experiment at the LHC in 2012 and corresponding to an integrated luminosity of 19.7$\,\text {fb}^{-1}$. It is performed on ${\mathrm{t}}\overline{\mathrm{t}}$ events in which the leading jet includes all ${\mathrm{t}} \rightarrow \mathrm{b} \mathrm {W^+}\rightarrow \mathrm{b} \mathrm{q} \overline{\mathrm{q}} '$ decay products. The other top quark is required to decay through the semileptonic mode $\overline{{\mathrm{t}}} \rightarrow \overline{{\mathrm{b}}} \mathrm {W^{-}}\rightarrow \overline{{\mathrm{b}}} \ell \overline{\nu } _\ell$, where $\ell$ can be either an electron or muon. The use of charge-conjugate modes is implied throughout this article. The semileptonic top quark decay serves as a means for selecting ${\mathrm{t}}\overline{\mathrm{t}}$ events without biasing the $m_{\text {jet}}$ distribution from the fully hadronic top quark decay. The highly boosted top quark jets used in the measurement are defined through the Cambridge–Aachen (CA) jet-clustering algorithm [49, 50] with a distance parameter $R=1.2$ and $p_{\mathrm{T}} >400\,\text {GeV}$. The $m_{\text {jet}}$ distribution is unfolded to the particle level and compared to predictions from MC simulations. The measurement is also normalised to a fiducial-region total cross section defined below, and shows the expected sensitivity to the value of $m_{{\mathrm{t}}}$. An extraction of the value of $m_{{\mathrm{t}}}$ is performed to assess the overall sensitivity of the measurement.

## The CMS detector

The central feature of the CMS detector is a superconducting solenoid of 6$\text {\,m}$ internal diameter, providing a magnetic field of 3.8 $\text {\,T}$. A silicon pixel and strip tracker, a lead tungstate crystal electromagnetic calorimeter (ECAL), and a brass and scintillator hadron calorimeter (HCAL), each composed of a barrel and two endcap sections reside within the magnetic volume. In addition to the barrel and endcap detectors, CMS has extensive forward calorimetry. Muons are detected using four layers of gas-ionization detectors embedded in the steel flux-return yoke of the magnet. The inner tracker measures charged particle trajectories within the pseudorapidity range $|\eta | < 2.5$. A two-stage trigger system [51] is used to select for analysis $\mathrm {p}\mathrm {p}$ collisions of scientific interest. A more detailed description of the CMS detector, together with a definition of the coordinate system and relevant kinematic variables, can be found in Ref. [52].

## Event reconstruction

The CMS experiment uses a particle-flow (PF) event reconstruction [53, 54], which aggregates input from all subdetectors. This information includes charged particle tracks from the tracking system and energies deposited in the ECAL and HCAL, taking advantage of the granularity of the subsystems. Particles are classified as electrons, muons, photons, and charged and neutral hadrons. Primary vertices are reconstructed using a deterministic annealing filter algorithm [55]. The vertex with the largest sum in the associated track $p_{\mathrm{T}} ^2$ values is taken to be the primary event vertex.

Muons are detected and measured in the pseudorapidity range $|\eta | < 2.1$ using the information collected in the muon and tracking detectors [56]. Tracks from muon candidates must be consistent with a muon originating from the primary event vertex, and satisfy track-fit quality requirements [57].

Electrons are reconstructed in the range $|\eta | < 2.1$, by combining tracking information with energy deposits in the ECAL [58, 59]. Electron candidates are required to originate from the primary event vertex. Electrons are identified through the information on the energy distribution in their shower, the track quality, the spatial match between the track and electromagnetic cluster, and the fraction of total cluster energy in the HCAL. Electron candidates that are consistent with originating from photon conversions in the detector material are rejected.

Since the top quark decay products can be collimated at high values of top quark $p_{\mathrm{T}}$, no isolation requirements on the leptons are imposed in either the trigger or in the offline selections (see Sect. 4). The imbalance in event $\mathbf {p_{\mathrm{T}}}$ is quantified as the missing transverse momentum vector ${\mathbf {p}}_{\mathrm {T}}^{\text {miss}}$, defined as the projection on the plane perpendicular to the beams of the negative vector sum of the momenta of all PF candidates in the event. Its magnitude is referred to as $p_{\mathrm{T}} ^\text {miss}$.

The PF candidates are clustered into jets by using the FastJet 3.0 software package [60]. Charged hadrons associated with event vertices other than the primary event vertex are removed prior to jet clustering. Isolated leptons (either electron or muon) are not part of the input list for jet finding [53, 54]. Small-radius jets are clustered with the anti-$k_{\mathrm {T}}$ jet-clustering algorithm [61] with a distance parameter $R=0.5$ (AK5 jets). These small-radius jets are used at the trigger level, in the first steps of the event selection, and for the identification of jets coming from the hadronisation of $\mathrm{b}$ quarks. If a nonisolated lepton candidate is found within the angular distance ${\Delta R < 0.5}$ of an AK5 jet, its four-momentum is subtracted from that of the jet to avoid double counting of energy and ensure proper jet energy corrections. The angular distance is given by $\Delta R = \sqrt{\smash [b]{(\Delta \phi )^2 + (\Delta \eta )^2}}$, where $\Delta \phi$ and $\Delta \eta$ are the differences in azimuthal angle (in radians) and pseudorapidity, respectively, between the directions of the lepton and jet. Large-radius jets are obtained by using the CA jet-clustering algorithm [49, 50] with $R=1.2$ (CA12 jets). When a lepton candidate is found among the PF candidates clustered into a CA12 jet, its four-momentum is subtracted from that of the CA12 jet. In this paper, the unmodified term ”jet” will refer to the broad CA12 jets.

All jets could contain neutral particles from additional $\mathrm {p}\mathrm {p}$ collisions in the same or nearby beam crossings (pileup). This extra contribution is subtracted based on the average expectation of the pileup in the jet catchment area [62]. This is done by calculating a correction for the average offset energy density in each event as a function of the number of primary vertices [63, 64]. The AK5 jets are identified as originating from the fragmentation of a $\mathrm{b}$ quark with the combined secondary vertex algorithm (CSV) [65]. A tight operating point is used, which has a misidentification probability of 0.1% for tagging light-parton jets with an average $p_{\mathrm{T}}$ of about 80$\,\text {GeV}$, and an efficiency of about 50% for a heavy-flavour jet with $p_{\mathrm{T}}$ in the range 50–160$\,\text {GeV}$. Above 160$\,\text {GeV}$, the efficiency decreases gradually to about 30% for a $p_{\mathrm{T}}$ value of 400$\,\text {GeV}$  [65]. All jets are required to satisfy quality selections to minimize the impact of calorimeter noise and other sources of misidentified jets [66]. Events are also required to satisfy selection criteria to remove events with large values of $p_{\mathrm{T}} ^\text {miss}$ from calorimeter noise, as described in Ref. [67].

The jet mass $m_{\text {jet}}$ is calculated from the four-vectors $p_i$ of all *i* PF particles clustered into a jet:

1 $$\begin{aligned} m_{\text {jet}} ^2 = \Bigl ( \sum _{i~\text {in jet}} p_i \Bigr )^2 , \end{aligned}$$

where the pion mass is assigned to all charged hadrons. The reconstruction of $m_{\text {jet}}$ for CA12 jets is studied by using a sample of highly boosted $\mathrm {W}\rightarrow \mathrm{q} \overline{\mathrm{q}} '$ decays merged into a single jet, as described in Sect. 5.5.

## Trigger and data

The data were recorded by using single-lepton triggers with no isolation requirement applied to the leptons. Events in the muon+jets channel use a trigger that requires at least one muon with $p_{\mathrm{T}} > 40$ $\,\text {GeV}$ and $|\eta |<2.1$. The efficiency for this trigger, measured in a ${\mathrm{Z}} \rightarrow \mu ^+\mu ^-$ sample, is 95% for muons measured within $|\eta |<0.9$, 85% for muons within $0.9<|\eta |<1.2$, and 83% for $1.2<|\eta |<2.1$.

The trigger for the electron+jets channel requires at least one electron with $p_{\mathrm{T}} > 30$ $\,\text {GeV}$ in conjunction with two AK5 jets that have $p_{\mathrm{T}} >100$ and $> 25$ $\,\text {GeV}$, for the leading and next-to-leading AK5 jet, respectively. Events are also included if triggered by a single AK5 jet with $p_{\mathrm{T}} >320\,\text {GeV}$. The additional events obtained through this single-jet trigger often contain an electron merged into a jet that cannot be resolved at the trigger stage. The resulting combined trigger efficiency is 90% for events with a leading AK5 jet with $p_{\mathrm{T}} <320\,\text {GeV}$. Above this value, the trigger has a turn-on behaviour and is fully efficient above a value of $350\,\text {GeV}$. The trigger efficiencies are measured in data and simulation using a tag-and-probe method in ${\mathrm{Z}}$/$\gamma ^*(\rightarrow \ell \ell )$+jets and dileptonic ${\mathrm{t}}\overline{\mathrm{t}}$ events. Small differences between data and simulation are corrected for by applying scale factors to the simulated events.

Top quark events, produced via the strong and electroweak interactions, are simulated with the next-to-leading-order (NLO) generator powheg 1.380 [68–72] with a value of $m_{{\mathrm{t}}} =172.5\,\text {GeV}$. The $\mathrm {W}(\rightarrow \ell \nu )$+jets and ${\mathrm{Z}}/\gamma ^*(\rightarrow \ell \ell )$+jets processes are simulated with MadGraph 5.1.5.11 [73], where Madspin [74] is used for the decay of heavy resonances. Diboson production processes ($\mathrm {W}$ $\mathrm {W}$, $\mathrm {W}$ ${\mathrm{Z}}$, and ${\mathrm{Z}} {\mathrm{Z}}$) are simulated with pythia  6.424 [75]. Simulated multijet samples are generated in MadGraph, but constitute a negligible background. For the estimation of systematic uncertainties, additional ${\mathrm{t}}\overline{\mathrm{t}}$ samples are generated with mc@nlo v3.41 [76] or with MadGraph for seven values of $m_{{\mathrm{t}}}$ ranging from 166.5 to 178.5$\,\text {GeV}$.

All the samples generated in MadGraph and powheg are interfaced with pythia  6 for parton showering and fragmentation (referred to as MadGraph +pythia and powheg +pythia, respectively). The MLM algorithm [77] used in MadGraph is applied during the parton matching to avoid double counting of parton configurations. The MadGraph samples use the CTEQ6L [78] parton distribution functions (PDFs). The powheg ${\mathrm{t}}\overline{\mathrm{t}}$ sample uses the CT10 [79] PDFs, whereas the single top quark processes use the CTEQ6M [80] PDFs. The pythia 6 Z2* tune [81, 82] is used to model the underlying event. Top quark events produced with mc@nlo use the CTEQ6M PDF set and herwig 6.520 [83] for parton showering and fragmentation (mc@nlo +herwig). The default herwig tune is used to model the underlying event.

The normalisations of the simulated event samples are taken from the NLO calculations of their cross sections that contain the next-to-next-to-leading-logarithm (NNLL) soft-gluon resummations for single top quark production [84], the next-to-next-to-leading-order (NNLO) calculations for $\mathrm {W}(\rightarrow \ell \nu )$+jets and ${\mathrm{Z}}/\gamma ^*(\rightarrow \ell \ell )$+jets [85–87], and the NLO calculation for diboson production [88]. The normalisation of the ${\mathrm{t}}\overline{\mathrm{t}}$ simulation is obtained from QCD NNLO calculations, again including resummation of NNLL soft-gluon terms [89–95].

A detailed simulation of particle propagation through the CMS apparatus and detector response is performed with Geant4 v9.2 [96]. For all simulated samples, the hard collision is overlaid with simulated minimum-bias collisions. The resulting events are weighted to reproduce the pileup distribution measured in data. The same event reconstruction software is used for data and simulated events. The resolutions and efficiencies for reconstructed objects are corrected to match those measured in data [56, 58, 64, 65, 97].

## Cross section measurement

### Strategy

The measurement is carried out in the $\ell$+jets channel, which allows the selection of a pure ${\mathrm{t}}\overline{\mathrm{t}}$ sample because of its distinct signature at large top quark boosts. The measurement is based on choosing kinematic quantities that do not bias the $m_{\text {jet}}$ distribution from fully hadronic top quark decays. A bias would be introduced by, e.g. selecting the leading jet based on the number of subjets, or requiring a certain maximum value of the *N*-subjettiness [98, 99], as applied in common top quark tagging algorithms [100–104]. Such a selection would lead to a distinct three-prong structure of the jet and thus reject events with one quark being soft or collinear with respect to the momentum of the top quark decay.

The fiducial region chosen for this investigation is studied through simulations at the particle level (defined by all particles with lifetimes longer than $10^{-8}$ s). The exact selection is detailed below. It relies on having a highly boosted semileptonic top quark decay, where the lepton from $\mathrm {W}\rightarrow \ell \nu _\ell$ is close in $\Delta R$ to the jet from the hadronisation of the accompanying $\mathrm{b}$ quark ($\mathrm{b}$ jet). A second high-$p_{\mathrm{T}}$ jet is selected, which is assumed to originate from the fully hadronic top quark decay. A veto on additional jets is employed, which ensures that the fully hadronic decay is merged into a single jet. The jet veto is also beneficial for calculating higher-order terms, as it suppresses the size of nonglobal logarithms [105], which appear because of the sensitivity of the jet mass to radiation in only a part of the phase space [106]. The event selection at the reconstruction level is chosen to ensure high efficiency while reducing non-${\mathrm{t}}\overline{\mathrm{t}}$ backgrounds. Finally, the $m_{\text {jet}}$ distribution is unfolded for experimental effects and then compared to different MC predictions at the particle level. A measurement of the normalised $m_{\text {jet}}$ distribution is performed as well, where the normalisation is performed by using the total measured ${\mathrm{t}}\overline{\mathrm{t}}$ cross section in the fiducial phase-space region.

### Definition of the fiducial phase space 

The ${\mathrm{t}}\overline{\mathrm{t}}$ cross section as a function of the mass of the leading jet is unfolded to the particle level, correcting for experimental effects, with the fiducial phase space at the particle level defined through the selection described below.

As mentioned previously, the measurement is performed in the $\ell$+jets channel, where $\ell$ refers to an electron or muon from the $\mathrm {W}$ boson decay. The $\tau$ lepton decays are not considered as part of the signal. Leptons are required to be within $|\eta | < 2.1$ and have $p_{\mathrm{T}} >45\,\text {GeV}$. Jets are clustered by using the CA algorithm with a distance parameter $R = 1.2$ and required to have $|\eta | < 2.5$. The value of *R* is chosen to optimize the relationship between obtaining a sufficient number of events and maintaining a narrow width in the jet-mass distribution. The four-momentum of the leading lepton is subtracted from the four-momentum of a jet if the lepton is found within an angular range of ${\Delta R < 1.2}$ of the jet axis. Events are selected if at least one jet has $p_{\mathrm {T,1}} >400\,\text {GeV}$ and a second jet has $p_{\mathrm {T,2}} >150\,\text {GeV}$. The leading jet in $p_{\mathrm{T}}$ is assumed to originate from the ${\mathrm{t}} \rightarrow \mathrm {W}\mathrm{b} \rightarrow \mathrm{q} \overline{\mathrm{q}} ^\prime \mathrm{b}$ decay, merged into a single jet. Consequently, the second jet is considered to originate from the fragmented $\mathrm{b}$ quark of the semileptonic top quark decay. To select events with a highly boosted topology, a veto is employed on additional jets with $p_{\text {T,veto}} >150\,\text {GeV}$. The jet veto removes about 16% of the signal events, but increases the fraction of fully merged top quark decays to about 40%, where an event is called fully merged if the maximum distance in $\Delta R$ between the leading jet at the particle level and each individual parton from the fully hadronic top quark decay is smaller than 1.2.

Two additional selection criteria are introduced to ensure that the leading jet includes all particles from the fully hadronic top quark decay. The angular difference $\Delta R(\ell , \text {j}_2)$ between the lepton and the second jet has to be smaller than 1.2. This, together with the veto on additional jets, ensures that the top quarks are produced back-to-back in the transverse plane. In addition, the invariant mass of the leading jet has to be greater than the invariant mass of the combination of the second jet and the lepton, $m_{\text {jet},1} > m_{\text {jet},2+\ell }$. This improves the choice of the leading jet as originating from the fully hadronic top quark decay.

**Fig. 1:**
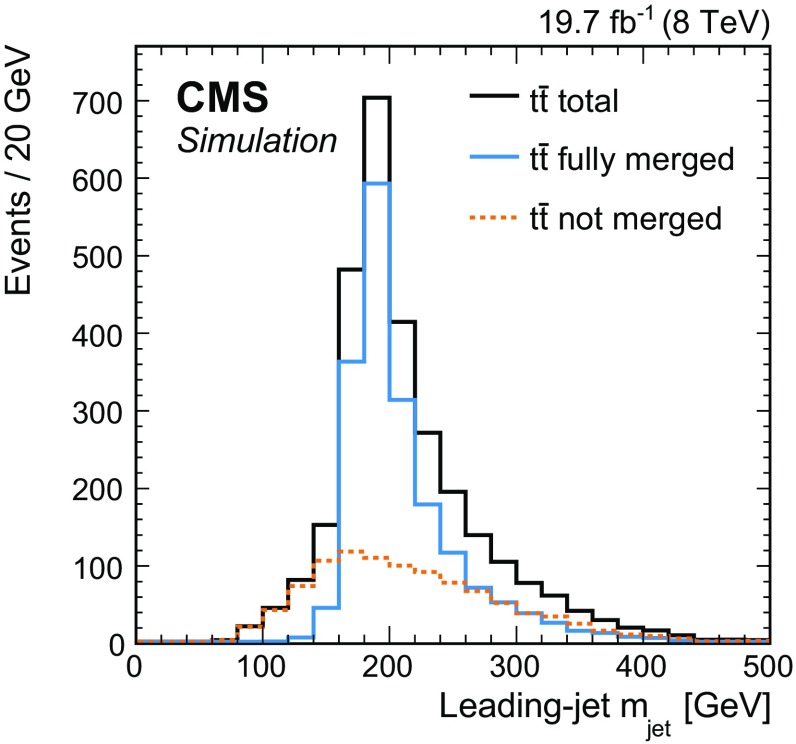
Simulated mass distributions of the leading jet in ${\mathrm{t}}\overline{\mathrm{t}}$ events for the $\ell$+jets channel at the particle level. The events are generated with powheg +pythia, and normalised to the integrated luminosity of the data. The distribution for the total number of selected events (*dark solid line*) is compared to events where the leading jet originates from the fully hadronic top quark decay (*light solid line*, “fully merged”), and to events where the leading jet does not include all the remnants (dotted line, “not merged”) from the fully hadronic top quark decay

The simulated distribution of the jet mass at the particle level after this selection is shown in Fig. 1. The distribution of all jets passing the particle-level selection is compared to distributions in jet mass from fully merged and not merged ${\mathrm{t}}\overline{\mathrm{t}}$ decays. After the selection outlined above, jets that do not originate from fully merged top quark decays with a fully hadronic final state are expected to constitute about 35% of all jets in the final data sample, as determined by using the powheg +pythia simulation.

### Selection of events at the reconstruction level

A selection is applied at the reconstruction level to obtain an enriched ${\mathrm{t}}\overline{\mathrm{t}}$ sample with high-$p_{\mathrm{T}}$ top quarks, based on leptons without an isolation requirement. As a second step, high-$p_{\mathrm{T}}$ jets are required to be kinematically similar to those selected at the particle level. Comparable kinematic properties between the reconstruction and particle levels lead to small bin-to-bin migrations and therefore to small corrections when unfolding the data.

Selected events must contain exactly one muon or electron with $p_{\mathrm{T}} >45\,\text {GeV}$ and $|\eta |<2.1$. Events with more than one lepton are vetoed to suppress contributions from dileptonic ${\mathrm{t}}\overline{\mathrm{t}}$ decays. To select highly boosted ${\mathrm{t}}\overline{\mathrm{t}}$ events, at least one AK5 jet is required to have $p_{\mathrm{T}} >150\,\text {GeV}$ and another AK5 jet $p_{\mathrm{T}} > 50\,\text {GeV}$, where both jets have to fulfil $|\eta |<2.4$. The suppression of background from multijet production is accomplished by using a two-dimensional (2D) isolation variable that is efficient at large top quark boosts, yet notably reduces multijet background. This 2D isolation requires the angular difference between the lepton and the nearest AK5 jet directions $\Delta R_{\text {min}}(\text {lepton, jets})$ to be greater than 0.5, or the perpendicular component of the lepton momentum relative to the nearest AK5 jet $p_{\text {rel,T}}$ to be larger than $25\,\text {GeV}$. In the calculation of these quantities, only AK5 jets with $p_{\mathrm{T}} >25\,\text {GeV}$ are considered. The efficiency of the 2D isolation requirement has been studied in data and simulation by using ${\mathrm{Z}}/\gamma ^*(\rightarrow \ell \ell )$+jets events [26].

A requirement on $p_{\mathrm{T}} ^\text {miss} >20\,\text {GeV}$ and on the scalar sum $p_{\mathrm{T}} ^\text {miss} +p_{\mathrm{T}} ^\ell > 150\,\text {GeV}$ reduces the contribution from multijet and ${\mathrm{Z}}/\gamma ^*(\rightarrow \ell \ell )$+jets production, where $p_{\mathrm{T}} ^\ell$ is the lepton transverse momentum. Given the presence of two $\mathrm{b}$ quarks in the events, at least one AK5 jet is required to be identified as originating from the fragmentation of a $\mathrm{b}$ quark by using the CSV algorithm, which reduces the contribution from $\mathrm {W}$+jets production. The electron channel includes an additional topological selection criterion to suppress the remaining residual contribution from multijet production:

$$\begin{aligned} | \Delta \phi ( \{ \mathrm {e}\,\text {or}\, \text {jet} \} , \,{\mathbf {p}}_{\mathrm {T}}^{\text {miss}}) - 1.5 | < p_{\mathrm{T}} ^\text {miss}/ 50\,\text {GeV}, \end{aligned}$$

with $\Delta \phi$ measured in radians and $p_{\mathrm{T}} ^\text {miss}$ in $\,\text {GeV}$. This criterion rejects events in which ${\mathbf {p}}_{\mathrm {T}}^{\text {miss}}$ points along the transverse momentum vector of the leading jet or the lepton. After these requirements, the background contribution from multijet production is negligible.

The selection procedure outlined above results in a ${\mathrm{t}}\overline{\mathrm{t}}$ sample with high purity and selection efficiency at large top quark $p_{\mathrm{T}}$. In addition, events are selected with kinematic requirements similar to those at the particle level. For each event to pass the selection, at least one jet is required with $p_{\mathrm{T}} >400\,\text {GeV}$ and another with $p_{\mathrm{T}} > 150\,\text {GeV}$, where both jets have to fulfil $|\eta |<2.5$. Contributions from not fully merged ${\mathrm{t}}\overline{\mathrm{t}}$ events are suppressed with a veto on additional jets with transverse momentum $p_{\mathrm{T}} > 150\,\text {GeV}$ and $|\eta | < 2.5$. The jet veto has an efficiency of $93\%$ for fully-merged signal events. The fraction of fully merged events with a back-to-back topology is further enhanced by selecting events with an angular difference $\Delta R(\ell , \text {j}_2)<1.2$ between the directions of the lepton and the subleading jet. To ensure that the leading jet originates from the fully merged top quark decay, its invariant mass is required to be larger than the mass of the subleading jet. With these selection criteria, the reconstruction efficiency for ${\mathrm{t}}\overline{\mathrm{t}}$ events where one top quark decays semileptonically in the fiducial region of the measurement is 23.2%. Several of the above criteria are relaxed in the unfolding procedure to define sideband regions included as additional bins in the response matrix, increasing thereby the reconstruction efficiency.

After the selection procedure, the contribution of non-signal ${\mathrm{t}}\overline{\mathrm{t}}$ events from ${\mathrm{t}}\overline{\mathrm{t}}$ decays to the $\tau$+jets, dilepton, and all-jets channels constitute, respectively, 7.3, 11.6, and $0.4\%$ of the selected events. These contributions are accounted for in the unfolding.

**Fig. 2:**
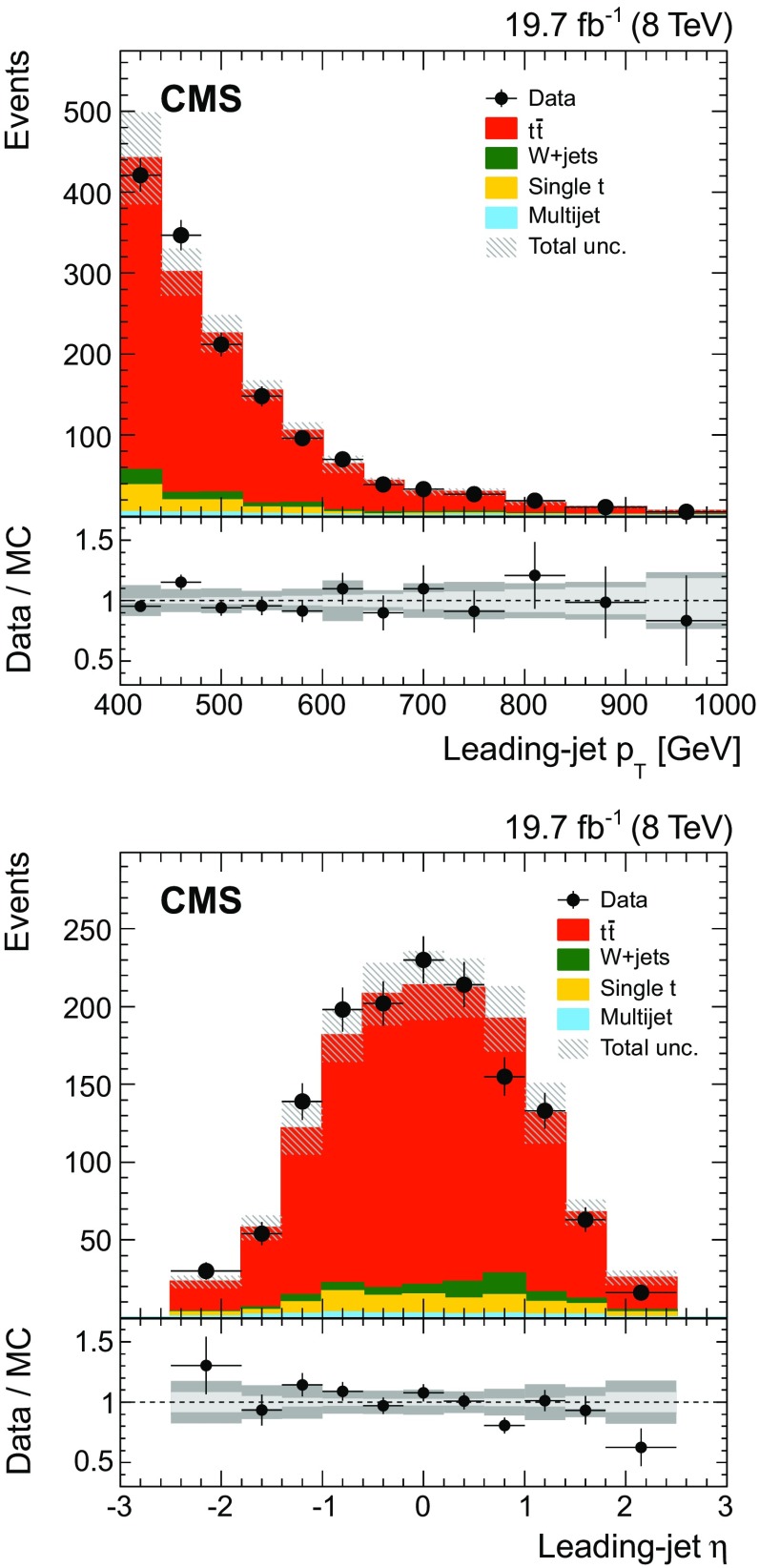
Distributions of $p_{\mathrm{T}}$ (*upper*) and $\eta$ (*lower*) of the leading jet from data (*points*) and simulation (*filled histograms*). The *vertical bars on the points* show the statistical uncertainty and the *horizontal bars* show the bin widths. The electron and muon channels are shown combined. The *hatched region* shows the total uncertainty in the simulation, including the statistical and experimental systematic uncertainties. The *panels* below show the ratio of the data to the simulation. The uncertainty *bands* include the statistical and experimental systematic uncertainties, where the statistical (*light grey*) and total (*dark grey*) uncertainties are shown separately in the ratio

**Fig. 3:**
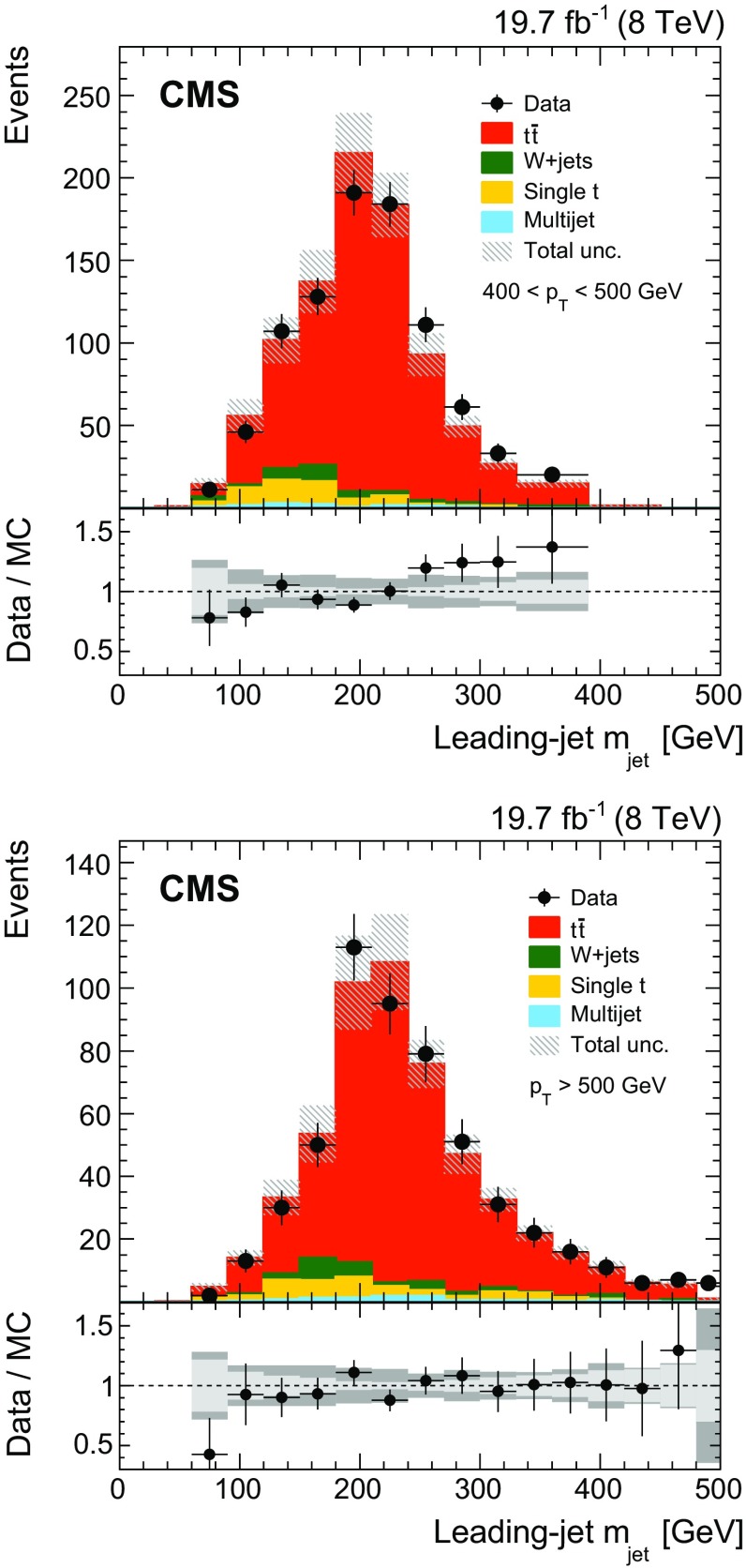
Distributions of the leading-jet invariant mass from data (*points*) and simulation (*filled histograms*). The *vertical bars on the points* show the statistical uncertainty and the *horizontal bars* show the bin widths for the combined electron and muon channels. The distributions for $p_{\mathrm{T}}$ bins of $400< p_{\mathrm{T}} < 500\,\text {GeV}$ (*upper*) and $p_{\mathrm{T}} > 500\,\text {GeV}$ (*lower*) are given. The *hatched region* shows the total uncertainty in the simulation, including the statistical and experimental systematic uncertainties. The *panels* below show the ratio of the data to the simulation. The uncertainty *bands* include the statistical and experimental systematic uncertainties, where the statistical (*light grey*) and total (*dark grey*) uncertainties are shown separately in the ratio

The distributions in $p_{\mathrm{T}}$ and $\eta$ for the leading jet in selected events are shown in Fig. 2 from data and simulation. The mass distribution of the leading jet at the reconstruction level is shown in Fig. 3 for the $p_{\mathrm{T}}$ regions of $400< p_{\mathrm{T}} < 500\,\text {GeV}$ (upper) and $p_{\mathrm{T}} > 500\,\text {GeV}$ (lower). In these plots the ${\mathrm{t}}\overline{\mathrm{t}}$ simulation is scaled such that the number of simulated events matches the number of selected events observed in data. Overall good agreement between data and the predictions is observed. The slight slope in the data/MC ratio of the jet mass distribution in Fig. 3 (upper) is covered by the jet energy and mass scale uncertainties, as described below.

Table 1 shows the total number of events observed in data together with the total number of signal and background events determined from simulation.

**Table 1:** Number of events obtained after applying the full selection. The results are given for the individual sources of background, ${\mathrm{t}}\overline{\mathrm{t}}$ signal, and data. The uncertainties correspond to the statistical and systematic components added in quadrature

Source	Number of events
Multijet	21 ± 21
$\mathrm {W}$+jets	60 ± 13
Single top quark	90 ± 21
Total background	171 ± 32
${\mathrm{t}}\overline{\mathrm{t}}$ signal	1410 ± 152
Data	1434

### Unfolding from the reconstruction level to the particle level

The transformation from the reconstruction to the particle level is carried out through a regularised unfolding based on a least-squares fit, implemented in the TUnfold [107] framework. This procedure suppresses the statistical fluctuations by a regularisation with respect to the count in each bin. The optimal regularisation strength is determined through a minimization of the average global correlation coefficient of the output bins [108]. Contributions from background processes such as $\mathrm {W}$+jets, single top quark, and multijet production are determined from simulation and subtracted from the data prior to the unfolding. Non-signal ${\mathrm{t}}\overline{\mathrm{t}}$ events are accounted for in the unfolding by including them in the response matrix, described below.

The response matrix is evaluated by using ${\mathrm{t}}\overline{\mathrm{t}}$ events simulated with ${\textsc {powheg}} {}+{\textsc {pythia}}$. It is obtained for the two regions in the leading-jet $p_{\mathrm{T}}$ of $400< p_{\mathrm{T}} < 500\,\text {GeV}$ and $p_{\mathrm{T}} >500\,\text {GeV}$. This division is needed to account for the distribution of the $p_{\mathrm{T}}$ spectrum. The response matrix includes three additional sideband regions to account for migrations in and out of the phase-space region of the measurement. These are obtained for a lower leading-jet $p_{\mathrm{T}}$ of $300< p_{\mathrm{T}} < 400 \,\text {GeV}$, a lower second-leading-jet $p_{\mathrm{T}}$ of $100< p_{\mathrm{T}} < 150 \,\text {GeV}$, and a higher veto-jet $p_{\mathrm{T}}$ of $150< p_{\mathrm{T}} < 200 \,\text {GeV}$. Events that are reconstructed, but do not pass the particle-level selections, are also included in the response matrix. The electron and muon channels are combined, and the combined distribution is unfolded to ensure a sufficient number of events in the unfolding procedure. The electron and muon channels are also unfolded separately, and the results are compared to verify their consistency.

### Uncertainties 

#### Statistical uncertainties

Statistical uncertainties in the unfolding procedure arise from three sources. The dominant source reflects the statistical fluctuations in the input data. Second are the uncertainties from the finite number of simulated events used to calculate the response matrix. The third source reflects the statistical uncertainties in the simulation of the background processes. After the unfolding, a total statistical uncertainty is obtained for each bin of the $m_{\text {jet}}$ distribution that includes the effects from all three sources, which are correlated among the individual measurement bins.

#### Experimental systematic uncertainties

Systematic uncertainties related to experimental effects are evaluated by changing calibration factors and corrections to efficiencies within their corresponding uncertainties. The resulting covariance matrix of the unfolded measurement is computed through standard error propagation. The uncertainties are evaluated by unfolding pseudo-data simulated with MadGraph +pythia. Pseudo-data are preferred over data because of the smaller statistical fluctuations in the estimation of the systematic uncertainties. The change in each parameter that yields the largest variation in the unfolded measurement is taken as the uncertainty owing to that parameter. The following sources of experimental systematic uncertainties are considered.

The applied jet energy corrections (JEC) depend on the $p_{\mathrm{T}}$ and $\eta$ of the individual jets. The JEC are obtained by using anti-$k_{\mathrm {T}}$ jets with $R=0.7$ (AK7) [64], and their use is checked on CA12 jets by using simulated events. Residual differences between generated and reconstructed jet momenta caused by the larger jet size used in this analysis result in increased uncertainties in the JEC by factors of two to four with respect to the AK7 values. Changes of the JEC within their uncertainties are made in the three-momenta of the jets to estimate the effect on the measured cross section. The jet mass is kept fixed to avoid double-counting of uncertainties when including the uncertainty in the jet-mass scale. A smearing is applied in the jet energy resolution (JER) as an $\eta$-dependent correction to all jets in the simulation. The corrections are again changed within their uncertainty to estimate the systematic uncertainty related to the JER smearing. The uncertainties are found to be small compared to the ones from the JEC. The jet-mass scale and the corresponding uncertainty in the CA12 jets have been studied in events that contain a $\mathrm {W}\rightarrow \mathrm{q} \overline{\mathrm{q}} '$ decay reconstructed as a single jet in ${\mathrm{t}}\overline{\mathrm{t}}$ production. The ratio of the reconstructed jet-mass peak positions in data and simulation is $1.015 \pm 0.012$. No correction to the jet-mass scale is applied, but an uncertainty of $1.5\%$ is assigned, corresponding to the difference in peak positions. The widths of the jet mass distributions are about 15$\,\text {GeV}$, consistent between data and simulation.

Corrections in $\mathrm{b}$ tagging efficiency are applied as $p_{\mathrm{T}}$-dependent scale factors for each jet flavour. The corresponding systematic uncertainties are obtained by changing the scale factors within their uncertainties. Pileup correction factors are applied to match the number of primary interactions to the instantaneous luminosity profile in data. The uncertainty is obtained by changing the total inelastic cross section by ${\pm }5\%$ [109]. Trigger and lepton identification scale factors are used to correct for differences in the lepton selection efficiency between data and simulation. The corresponding uncertainties are computed by changing the scale factors within their uncertainties [56, 58].

#### Normalisation uncertainties

The effects from uncertainties in background processes are calculated by changing the amount of background subtracted prior to the unfolding and propagating the effect to the output. The uncertainty in the $\mathrm {W}$+jets cross section is taken to be 19%, as obtained from a measurement of $\mathrm {W}$+heavy-flavour quark production [110]; an uncertainty of 23% is applied to the single top quark cross section [111]; and an uncertainty of 100% is assumed for multijet production, estimated from the comparison of various kinematic distributions between data and simulation. Uncertainties affecting the overall normalisation are added in quadrature to the total uncertainty after the unfolding. An uncertainty of 2.6% is applied subsequently for the integrated luminosity [112].

#### Modelling uncertainties

The unfolding is checked for its dependence on the simulation of ${\mathrm{t}}\overline{\mathrm{t}}$ production through the use of alternative programs to generate events. The effect on the measurement is estimated by using one simulation as pseudo-data input to the unfolding, and another for the calculation of the response matrix. The unfolded result is then compared to the particle-level distribution from the simulation used as pseudo-data. Differences between the unfolded result and the truth-level distribution are taken as the modelling uncertainties.

The uncertainty from the choice of MC generator is estimated by unfolding pseudo-data simulated with MadGraph +pythia through a response matrix evaluated with powheg +pythia. The effect from the choice of the parton-shower simulation is estimated from events generated with mc@nlo +herwig.

The dependence on the choice of $m_{{\mathrm{t}}}$ in the simulation used to correct the data is also checked. While the unfolded measurement is largely independent of the choice of $m_{{\mathrm{t}}}$, residual effects from the kinematic properties of the leptons and jets can lead to additional uncertainties. These uncertainties are evaluated by using events simulated with MadGraph +pythia for seven values of $m_{{\mathrm{t}}}$ from 166.5 to 178.5$\,\text {GeV}$, as pseudo-data. This range is considered because no measurement of $m_{{\mathrm{t}}}$ in this kinematic regime exists, and a stable result, independent of the specific choice of $m_{{\mathrm{t}}}$, is therefore crucial. For this check, the response matrix is obtained with MadGraph +pythia and a value of $m_{{\mathrm{t}}} = 172.5\,\text {GeV}$. The envelope of the uncertainty obtained for different values of $m_{{\mathrm{t}}}$ is used to define an additional modelling uncertainty.

The uncertainty from the uncalculated higher-order terms in the simulation is estimated by changing the choice of the factorisation and renormalisation scales $\mu _\mathrm {F}$ and $\mu _\mathrm {R}$. For this purpose events simulated with powheg +pythia are used, where the scales are changed up and down by factors of two relative to their nominal value. This is set to $\mu _\mathrm {F}^2 = \mu _\mathrm {R}^2 = Q^2$, where the scale of the hard process is defined by $Q^2 = m_{{\mathrm{t}}} ^2 + \sum p_{\mathrm{T}} ^2$ with the sum over all additional final-state partons in the matrix-element calculation. Events with varied scales are unfolded through a response matrix obtained with the nominal choice of scales. The uncertainty in the measurement is defined by the largest change found in the study.

Uncertainties from the PDF are evaluated by using the eigenvectors of the CT10 PDF set with the powheg +pythia simulation. The resulting differences in the response matrix are propagated to the measurement. The individual uncertainties for each eigenvector are scaled to the 68% confidence level and added in quadrature [79].

#### Summary of uncertainties

**Fig. 4:**
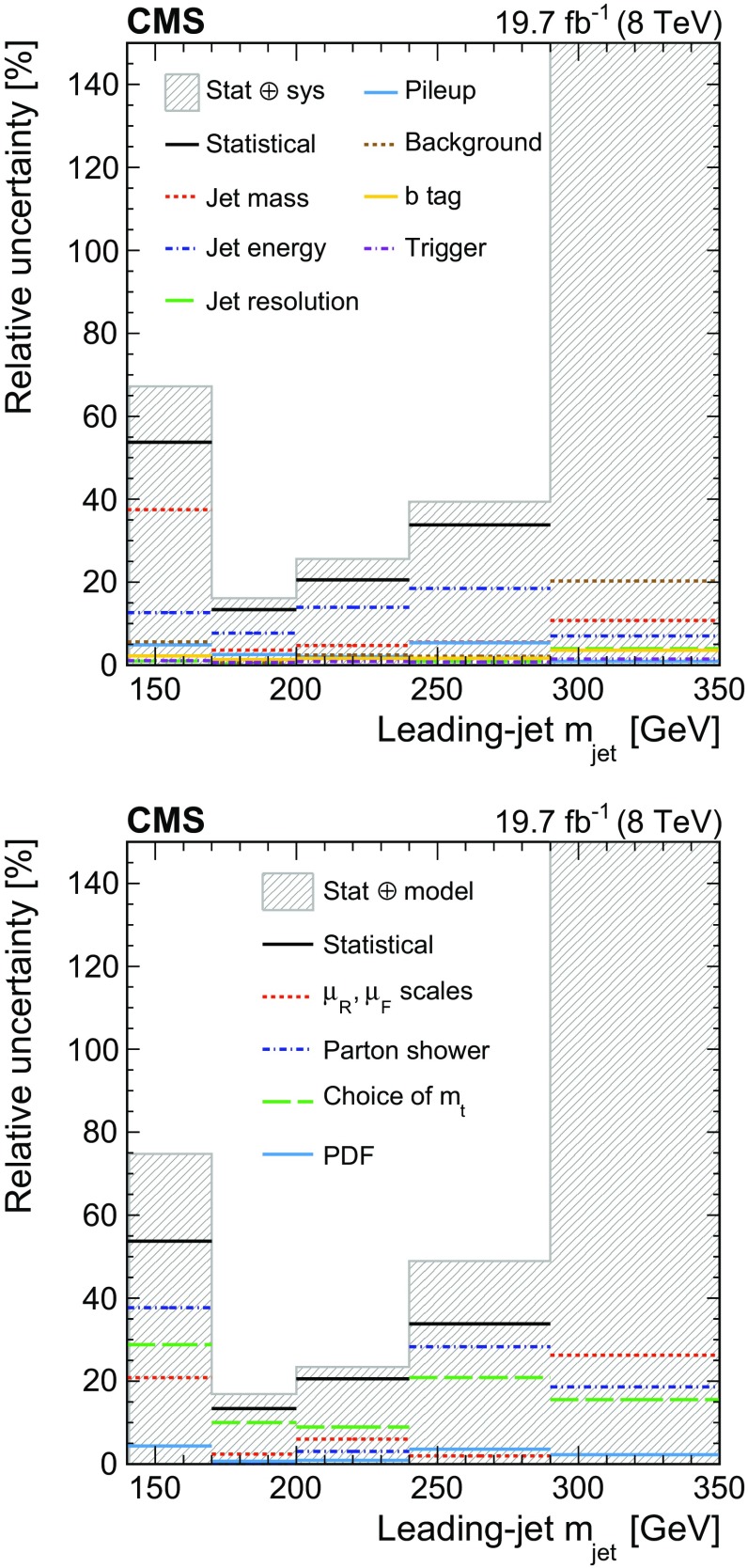
Statistical uncertainties compared to the individual experimental systematic uncertainties (*upper*), and statistical uncertainties compared to the systematic uncertainties originating from the modelling of ${\mathrm{t}}\overline{\mathrm{t}}$ production (*lower*), as a function of the leading-jet mass. The total uncertainties are indicated by the *grey cross-hatched regions*. The statistical and total uncertainties in the last bin are around 300% and exceed the vertical scale. The size of the horizontal bars represents the bin widths

A summary of the relative uncertainties in this measurement is shown in Fig. 4. The largest contribution is from the statistical uncertainties. The experimental systematic uncertainties are far smaller than those from the modelling of ${\mathrm{t}}\overline{\mathrm{t}}$ production. The largest uncertainties are expected to improve considerably with more data at higher centre-of-mass energies. Besides a reduction of the statistical uncertainties, an unfolding of the data using finer bins and as a function of more variables will then be possible, which will result in a reduction of the systematic uncertainties from the simulation of ${\mathrm{t}}\overline{\mathrm{t}}$ events. More data will also allow for a measurement that uses smaller jet sizes, which will reduce the uncertainties coming from the jet energy and jet mass scales.

### Cross section results 

**Table 2:** Summary of the selection criteria used to define the fiducial region of the measurement

Leptons	$p_{\mathrm{T}} ^\ell >45\,\text {GeV}$		$|\eta ^\ell | < 2.1$
Jets	$p_{\mathrm {T,1}} >400\,\text {GeV}$	$\Bigg \}$	$|\eta | < 2.5$
	$$p_{\mathrm {T,2}} >150\,\text {GeV} $$		
	$$p_{\text {T,veto}} >150\,\text {GeV} $$		
Event	$\Delta R(\ell , \text {j}_2)<1.2$		
	$$m_{\text {jet},1} > m_{\text {jet},2+\ell }$$		

The particle-level ${\mathrm{t}}\overline{\mathrm{t}}$ cross section for the fiducial phase-space region is measured differentially as a function of the leading-jet mass in the $\ell$+jets channel. The selection criteria defining the fiducial measurement region are summarised in Table 2 (cf. Sect. 5.2).

**Fig. 5:**
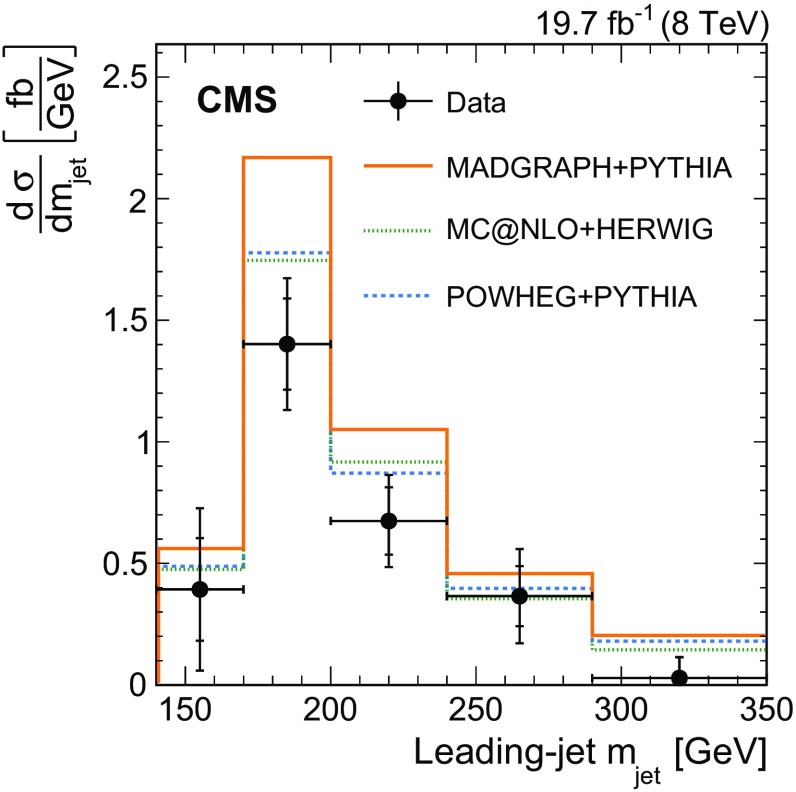
Fiducial-region particle-level differential ${\mathrm{t}}\overline{\mathrm{t}}$ cross sections as a function of the leading-jet mass. The cross sections from the combined electron and muon channels (*points*) are compared to predictions from the MadGraph +pythia, powheg +pythia, and mc@nlo +herwig generators (*lines*). The vertical bars represent the statistical (*inner*) and the total (*outer*) uncertainties. The *horizontal bars* show the bin widths

**Table 3:** Measured particle-level ${\mathrm{t}}\overline{\mathrm{t}}$ differential cross sections in the fiducial region as a function of $m_{\text {jet}}$, with the individual and total uncertainties in percent

Range in $m_{\text {jet}}$ ($\text {GeV}$ )	140–170	170–200	200–240	240–290	290–350
Integrated cross section (fb)	12	42	27	18	1.7
Statistical uncertainty (%)	54	13	21	34	300
Systematic uncertainty (%)	40	9	16	20	25
Model uncertainty (%)	52	10	11	35	36
Total uncertainty (%)	85	19	28	53	300

The measured differential cross section as a function of the leading-jet mass in this fiducial region is shown in Fig. 5, and the numerical values are given in Table 3. The full covariance matrices are given in Appendix A. The data are compared to simulated distributions obtained with powheg +pythia, MadGraph +pythia, and mc@nlo +herwig. The total measured ${\mathrm{t}}\overline{\mathrm{t}}$ cross section for $140< m_{\text {jet}} < 350\,\text {GeV}$ in the fiducial region is $\sigma = 101 \pm 11\,\text {(stat)} \pm 13\,\text {(syst)} \pm 9\,(\text {model})\text {\,fb}$, where the last uncertainty is from the modelling of the ${\mathrm{t}}\overline{\mathrm{t}}$ signal. Combining all the uncertainties in quadrature gives a value of $\sigma = 101 \pm 19\text {\,fb}$. The predicted fiducial-region cross sections from the MadGraph +pythia and powheg +pythia ${\mathrm{t}}\overline{\mathrm{t}}$ simulations, assuming a total ${\mathrm{t}}\overline{\mathrm{t}}$ cross section of 253 $\text {\,pb}$  [89–95], are $159\,^{+17}_{-18}$ and $133\,^{ +18}_{ -28}\text {\,fb}$, respectively, where the uncertainties are systematic and come from the variations of $\mu _\mathrm {R}$ and $\mu _\mathrm {F}$. The predictions exceed the measurements, consistent with previously measured ${\mathrm{t}}\overline{\mathrm{t}}$ cross sections at large top quark $p_{\mathrm{T}}$  [16, 17]. A similar trend is observed when comparing the data to the prediction from mc@nlo +herwig. Recent NNLO calculations [113] of the top quark $p_{\mathrm{T}}$ spectrum alleviate this discrepancy.

**Fig. 6:**
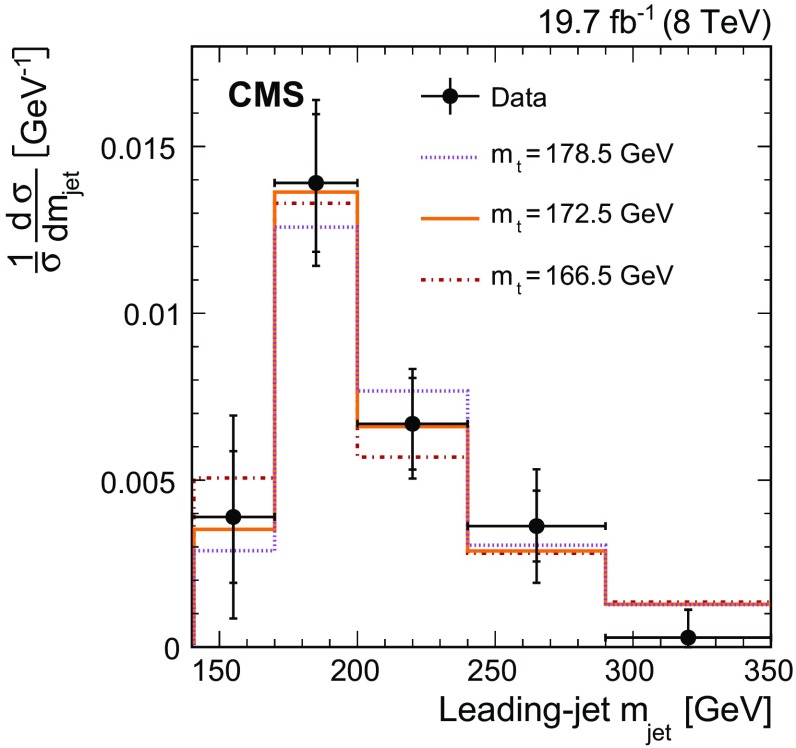
The normalised particle-level ${\mathrm{t}}\overline{\mathrm{t}}$ differential cross section in the fiducial region as a function of the leading-jet mass. The measurement is compared to predictions from MadGraph +pythia for three values of $m_{{\mathrm{t}}}$. The *vertical bars* represent the statistical (*inner*) and the total (*outer*) uncertainties. The *horizontal bars* show the bin widths

The normalised differential cross section $(1/\sigma ) (\mathrm{d}\sigma /\mathrm{d}m_{\text {jet}} {})$ is obtained by dividing the differential cross sections by the total cross section in the $m_{\text {jet}}$ range from 140 to 350$\,\text {GeV}$. The result is shown in Fig. 6, together with the predictions of MadGraph +pythia for three values of $m_{{\mathrm{t}}}$. The numerical values of the measured particle-level cross sections are given in Table 4, together with the individual and total uncertainties. The covariance matrices of the measurement are given in Appendix A. The data are well described by the simulation, showing that the overall modelling of the top quark jet mass is acceptable, once the disagreement with the total cross section at large $p_{\mathrm{T}}$ is eliminated by the normalisation. The sensitivity of the measurement to $m_{{\mathrm{t}}}$ is clearly visible, albeit compromised by the overall uncertainties.

**Table 4:** Values of the particle-level ${\mathrm{t}}\overline{\mathrm{t}}$ differential cross section in the fiducial region, normalized to unity, as a function of the leading-jet mass. The individual and total uncertainties are given in percent

Range in $m_{\text {jet}}$ ($\text {GeV}$ )	140–170	170–200	200–240	240–290	290–350
Integrated normalised cross section	0.12	0.42	0.27	0.18	0.017
Statistical uncertainty (%)	51	15	21	29	290
Systematic uncertainty (%)	34	5	9	13	27
Model uncertainty (%)	48	9	10	34	36
Total uncertainty (%)	78	18	25	47	300

## Sensitivity to the top quark mass

Calculations of $m_{\text {jet}}$ for ${\mathrm{t}}\overline{\mathrm{t}}$ production from first principles, by using a well-defined definition of $m_{{\mathrm{t}}}$ and not relying on parton shower and hadronisation models, are not yet available for the LHC. Still, a determination of the top quark mass parameter in general-purpose event generators that uses the normalised particle-level cross sections provides a proof of principle for the feasibility of the method, a cross-check on other determinations of $m_{{\mathrm{t}}}$, and an estimate of the current measurement’s sensitivity. The value of $m_{{\mathrm{t}}}$ is determined from the normalised differential cross section measurements given in Table 4, since only the shape of the $m_{\text {jet}}$ distribution can be reliably calculated. Correlations are taken into account through the full covariance matrix of the measurement, which is given in Appendix A. Theoretical predictions are obtained from MadGraph +pythia for different values of $m_{{\mathrm{t}}}$. A fit is performed based on the $\chi ^2$ evaluated as $\chi ^2 = d^T V^{-1} d$, where *d* is the vector of differences between the measured normalised cross sections and the predictions, and *V* is the covariance matrix, which includes the statistical, experimental systematic, modelling, and theoretical uncertainties. The latter are calculated by changing up and down by factors of two the scales $\mu _\mathrm {R}$ and $\mu _\mathrm {F}$ in the MadGraph +pythia simulation. The resulting uncertainties are added to the covariance matrix. The $\chi ^2$ values obtained for different values of $m_{{\mathrm{t}}}$ are fitted by a second-order polynomial to determine the minimum, and the uncertainty is determined by a change in $\chi ^2$ of 1.0. The result is

2 $$\begin{aligned} m_{{\mathrm{t}}} =&170.8 \pm 6.0\,\text {(stat)} \pm 2.8\,\text {(syst)} \end{aligned}$$

3 $$\begin{aligned}&\pm 4.6\,\text {(model)} \pm 4.0\,\text {(theo)} \,\text {GeV} \nonumber \\ =&170.8 \pm 9.0\,\text {GeV}, \end{aligned}$$

4 $$\begin{aligned} m_{{\mathrm{t}}}&= 170.8 \pm 6.0\,\text {(stat)} \pm 2.8\,\text {(syst)} \pm 4.6\,\text {(model)} \pm 4.0\,\text {(theo)} \,\text {GeV} \end{aligned}$$

5 $$\begin{aligned}&= 170.8 \pm 9.0\,\text {GeV}, \end{aligned}$$

where the total uncertainty in Eq. (3 5) is the sum in quadrature of the individual uncertainties in Eq. (2 4). The fit has a minimum $\chi ^2$ of 1.6 for three degrees of freedom. This measurement is the first determination of $m_{{\mathrm{t}}}$ from boosted ${\mathrm{t}}\overline{\mathrm{t}}$ production, calibrated to the MadGraph +pythia simulation. It is consistent with recent determinations of $m_{{\mathrm{t}}}$ that use MC event generators [33, 35–37], cross section measurements [6, 34, 114], and indirect constraints from electroweak fits [115].

## Summary and outlook

The first measurement of the differential ${\mathrm{t}}\overline{\mathrm{t}}$ cross section has been performed in the $\ell$+jets channel as a function of the leading-jet mass $m_{\text {jet}}$ in the highly boosted top quark regime. The measurement is carried out in a fiducial region with fully merged top quark decays in hadronic final states, corrected to the particle level. The normalised differential cross section as a function of $m_{\text {jet}}$ agrees with predictions from simulations, indicating the good quality of modelling the jet mass in highly boosted top quark decays. The total fiducial-region cross section for $m_{\text {jet}}$ between 140 and 350$\,\text {GeV}$ is measured to be $101 \pm 19\text {\,fb}$, which is below the predicted value. This difference is consistent with earlier measurements of a softer top quark $p_{\mathrm{T}}$ spectrum observed in data than in simulation [16, 17]. This measurement is a first step towards measuring unfolded jet substructure distributions in highly boosted top quark decays. A detailed understanding of these is crucial for measurements and searches for new physics making use of top quark tagging algorithms.

The peak position in the $m_{\text {jet}}$ distribution is sensitive to the top quark mass $m_{{\mathrm{t}}}$. This can be used for an independent determination of $m_{{\mathrm{t}}}$ in the boosted regime, with the prospect of reaching a more reliable correspondence between the top quark mass in any well-defined renormalisation scheme and the top quark mass parameter in general-purpose event generators.

The normalised particle-level ${\mathrm{t}}\overline{\mathrm{t}}$ differential cross section measurement as a function of $m_{\text {jet}}$ is used to extract a value of $m_{{\mathrm{t}}}$ in order to estimate the current sensitivity of the data. The value obtained, $m_{{\mathrm{t}}} = 170.8 \pm 9.0\,\text {GeV}$, is consistent with the current LHC and Tevatron average of $173.34 \pm 0.27\,\text {(stat)} \pm 0.71\,\text {(syst)} \,\text {GeV}$ [116], albeit with a much larger uncertainty.

New data at higher centre-of-mass energies and with larger integrated luminosities will lead to an improvement in the statistical uncertainty. More data can also lead to reductions in the experimental systematic uncertainties, most notably that from the jet mass scale, which is expected to improve with smaller jet distance parameters. In addition, improvements in the modelling uncertainty are expected because of stronger constraints on the simulation in the highly boosted regime. A reduction in the theoretical uncertainty is also foreseen with the emergence of higher-order calculations. The results obtained in this analysis show the feasibility of the method to obtain the top quark mass in the highly boosted regime. This can provide an important ingredient for studies of the relation between the value of the top quark mass obtained from MC event generators and the one obtained from first-principle calculations.

## A Covariance matrices

The covariance matrices that involve just the statistical components, and the ones involving the total uncertainty (i.e. the statistical, experimental systematic, and modelling uncertainties) are provided in this appendix. All experimental, as well as the PDF and parton-shower uncertainties, are treated as fully correlated in the calculation of the covariance matrices. The uncertainties in the renormalisation and factorisation scale include correlations in the first three bins, and the uncertainties coming from the choice of $m_{{\mathrm{t}}}$ are treated as uncorrelated. Bins 1 to 5 correspond to the following ranges in $m_{\text {jet}}$: 140–170, 170–200, 200–240, 240–290, and 290–350$\,\text {GeV}$. The covariance matrices for the differential $m_{\text {jet}}$ measurement are given in Tables 5 and 6 for the statistical and total uncertainties, respectively. The covariance matrices for the normalised measurement are given in Tables 7 and 8. Note that the covariance matrices of the normalised measurement are singular, and only four out of the five measurement bins are used in the determination of $m_{{\mathrm{t}}}$.

**Table 5:** Covariance matrix for the statistical uncertainties in the differential cross section. All entries are given in units of (fb$^2$)

Bin	1	2	3	4	5
1	+40.1	−4.3	−8.0	−0.2	−0.6
2		+31.7	−1.5	−8.1	+0.8
3			+30.7	+1.0	−4.5
4				+38.1	+7.3
5					+26.2

**Table 6:** Covariance matrix for the total uncertainties in the differential cross section, including all systematic and modelling uncertainties. All entries are given in units of (fb$^2$)

Bin	1	2	3	4	5
1	+100.4	+10.4	−0.3	−22.5	+1.6
2		+66.1	+11.0	+1.4	+0.8
3			+57.4	+12.0	−4.7
4				+93.8	+5.3
5					+26.7

**Table 7:** Covariance matrix for the statistical uncertainties in the normalised differential cross section. All entries are given in units of ($10^{-4}$)

Bin	1	2	3	4	5
1	+35.0	−11.2	−13.0	−6.7	−4.2
2		+38.3	+0.7	−17.2	−10.6
3			+30.1	−6.0	−11.8
4				+28.1	+1.8
5					+24.8

**Table 8:** Covariance matrix for the total uncertainties in the normalised differential cross section, including all systematic and modelling uncertainties. All entries are given in units of ($10^{-4}$)

Bin	1	2	3	4	5
1	+83.2	−18.9	−21.0	−40.7	−2.6
2		+55.5	−2.6	−23.7	−10.4
3			+43.1	−7.4	−12.0
4				+72.4	−0.5
5					+25.4
